# Comprehensive computational analysis reveals YXXΦ[I/L/M/F/V] motif and YXXΦ-like tetrapeptides across HFRS causing Hantaviruses and their association with viral pathogenesis and host immune regulation

**DOI:** 10.3389/fimmu.2022.1031608

**Published:** 2022-10-06

**Authors:** Fatima Noor, Usman Ali Ashfaq, Muhammad Asif, Muhammad Muzammal Adeel, Abdulrahman Alshammari, Metab Alharbi

**Affiliations:** ^1^ Department of Bioinformatics and Biotechnology, Government College University, Faisalabad, Pakistan; ^2^ Department of Environmental Health Science, College of Public Health, University of Georgia, Athens, GA, United States; ^3^ Department of Pharmacology and Toxicology, College of Pharmacy, King Saud University, Riyadh, Saudi Arabia

**Keywords:** hemorrhagic fever with renal syndrome, hantavirus, YXXΦ[I/L/M/F/V] motif, YXXΦ-like tetrapeptides, 3D structure, post-translational modifications

## Abstract

Hemorrhagic fever with renal syndrome (HFRS) is an acute zoonotic disease transmitted through aerosolized excrement of rodents. The etiology of HFRS is complex due to the involvement of viral factors and host immune and genetic factors. The viral species that dominantly cause HFRS are Puumala virus (PUUV), Seoul virus (SEOV), Dobrava-Belgrade virus (DOBV), and Hantaan virus (HTNV). Despite continuous prevention and control measures, HFRS remains a significant public health problem worldwide. The nucleocapsid protein of PUUV, SEOV, DOBV, and HTNV is a multifunctional viral protein involved in various stages of the viral replication cycle. However, the exact role of nucleoproteins in viral pathogenesis is yet to be discovered. Targeting a universal host protein exploited by most viruses would be a game-changing strategy that offers broad-spectrum solutions and rapid epidemic control. The objective of this study is to understand the replication and pathogenesis of PUUV, SEOV, DOBV, and HTNV by targeting tyrosine-based motif (YXXΦ[I/L/M/F/V]) and YXXΦ-like tetrapeptides. In the light of the current study, *in silico* analysis uncovered many different YXXΦ[I/L/M/F/V] motifs and YXXΦ-like tetrapeptides within nucleoproteins of PUUV, SEOV, DOBV, and HTNV. Following that, the 3D structures of nucleoproteins were predicted using AlphaFold2 to map the location of YXXΦ[I/L/M/F/V] motif and YXXΦ-like tetrapeptides in a 3D environment. Further, *in silico* analysis and characterization of Post Translational Modifications (PTMs) revealed multiple PTMs sites within YXXΦ[I/L/M/F/V] motif and YXXΦ-like tetrapeptides, which contribute to virulence and host immune regulation. Our study proposed that the predicted YXXΦ[I/L/M/F/V] motif and YXXΦ-like tetrapeptides may confer specific functions such as virulence, host immune regulation, and pathogenesis to nucleoproteins of PUUV, SEOV, DOBV, and HTNV. However, *in vivo* and *in vitro* studies on YXXΦ[I/L/M/F/V] motif and YXXΦ-like tetrapeptides will assign new biological roles to these antiviral targets.

## Introduction

Hemorrhagic fever with renal syndrome (HFRS) is a major rodent-borne zoonosis (1, 2). About 60,000–100,000 cases of human HFRS were reported annually in more than 70 countries around the world (3). HFRS is characterized by hypotension, fever, kidney damage, and bleeding. HFRS is an acute zoonotic viral disease and a distinct clinical syndrome endemic in Europe and Asia (4–6). The spatial heterogeneity of HFRS may be influenced by geographical differences and socioeconomic status (7, 8). Moreover, the outbreak of HFRS can occur in a short time and infect a large community of people (9, 10). Until now, effective clinical treatment is lacking for HFRS and consists of symptomatic and supportive care, mainly focusing on maintaining blood pressure and balancing fluid and electrolyte composition (11). Therefore, there is an urgent need to find an effective treatment for HFRS.

HFRS is caused by different species of hantaviruses including Puumala virus (PUUV) (12), Seoul virus (SEOV) (13), Dobrava-Belgrade virus (DOBV) (14), and Hantaan virus (HTNV) (15). Hantaviruses are membrane-enveloped viruses. Hantavirus is a genus of negative-sense, single-stranded, enveloped RNA viruses in the family Hantaviridae within the order Bunyavirales (16–18). The hantavirus genome is composed of a three segments coding for G1 and G2 glycoproteins, nucleocapsid protein, and viral polymerase. Comparatively to non-pathogenic strains, pathogenic hantaviruses significantly alter the transcriptional activity of many cellular genes (19, 20). Recent studies provide solid evidence that hantaviruses’ nucleocapsid proteins have a key role in virus transcription, replication, and assembly (21, 22). The nucleoprotein, encoded by the S segment, of hantaviruses consists of 429 to 433 amino acids (23). This nucleoprotein interacts with the host proteins and limits the activation of the major antiviral signaling pathways in affected cells. Furthermore, hantaviruses do not appear to have any pathogenic effects on their natural hosts. However, transmission to humans can result in deadly illnesses, particularly in South America, where the lethality rate of the hantavirus exceeds 40% (24, 25).

YXXΦ[I/L/M/F/V] is a tyrosine-based sorting motif in which Φ is an amino acid with bulky hydrophobic side chain, and X can be any residues (26). YXXΦ[I/L/M/F/V] motif was crucial for endocytosis from the cell surface and intracellular protein transport (27). YXXΦ[I/L/M/F/V] motif has a significant role in the internalization process and is typically found in the cytoplasmic domains of various transmembrane receptors, including the transferrin and asialoglycoprotein receptors (28). In the secretory and endosomal pathways, YXXΦ[I/L/M/F/V] signal motifs have dual specificity in the form of an endocytotic functional motif and a trafficking signal (29). Some viruses have incorporated these signaling motifs into their proteins and then utilize it to regulate the immune system (30–32). Karamichali et al. (33) reported that YXXΦ motif plays a pivotal role in the events following Hepatitis C virus (HCV) non-enveloped capsid-like particles clathrin-mediated endocytosis, which affects the HCV life cycle by enhancing viral replication.

The C terminus of Gn-cytoplasmic tail contains a highly conserved YxxL motif, and Hantavirus Cardiopulmonary Syndrome-causing hantaviruses are claimed to have two such motifs that form an immunoreceptor tyrosine-based activation motif (ITAM) (34). However, no study todate provide evidences about the existence of YXXΦ[I/L/M/F/V] motifs in HFRS-causing hantavirus. In the current work, YXXΦ[I/L/M/F/V] motif and YXXΦ-like tetrapeptides were mapped within nucleoprotein sequences of PUUV, SEOV, DOBV, and HTNV. To our knowledge, this is the first study that uncovered the putative YXXΦ[I/L/M/F/V] motif and YXXΦ-like tetrapeptides as well as their potential role in the host immune regulation, pathogenesis, and virulence. Moreover, these findings have sparked a new interest in the search treatment options for HFRS by targeting YXXΦ[I/L/M/F/V] motif and YXXΦ-like tetrapeptides. Additionally, we believe that targeting YXXΦ[I/L/M/F/V] motifs and YXXΦ-like tetrapeptides with pharmaceutical drugs that will result in PTMs with appropriate detrimental effects on immunological qualities and structural integrity of nucleoproteins, may contribute to design of effective anti-HFRS treatments.

## Material and methods

### Retrieval of proteins sequences and multiple sequence alignment

Retrieval of protein sequences is a preliminary step for identifying the tyrosine-based motifs to predict the host-viral interaction. The nucleoprotein sequences of Puumala virus (PUUV), Seoul virus (SEOV) Dobrava-Belgrade virus (DOBV), and Hantaan virus (HTNV) were retrieved from UniProt (35) with the following UniProt identifiers: P27313 (PUUV), W0LUE3 (SEOV), Q805Q9 (DOBV), and P05133 (HTNV) respectively. These sequences were then analyzed for putative YXXΦ[I/L/M/F/V] motifs and YXXΦ-like tetrapeptides.

Aligning multiple sequences assess highly conserved regions as compared to other regions. Thus, similar regions may help classify sequences or inform experiment design. Regarding this, the obtained nucleoprotein sequences of PUUV, SEOV, DOBV, and HTNV were then subjected to Clustal Omega (36) for Multiple Sequence Alignment (MSA) with default parameters (includes single distance matrix calculation along with clustering and one set of N‐1 pairwise alignments). Clustal Omega is MSA software that can accurately align thousands of sequences in a short time.

### Analysis of YXXΦ[I/L/M/F/V] motifs

The nucleoprotein sequences of PUUV, SEOV, DOBV, and HTNV were scanned with Eukaryotic Linear Motif (ELM) (37) web server for predicting the tyrosine-based motif (YXXΦ[I/L/M/F/V]) based on contextual information, including taxonomy, evolutionary conservation, cellular compartment, and structural features. ELM is a freely available repository of short linear motifs that have been experimentally verified and manually curated. The motif probability cutoff was set to 100 and other parameters were set to the default values. Extensive literature search was also made to map the YXXΦ[I/L/M/F/V] motif and YXXΦ-like tetrapeptides within nucleoprotein sequences.

### Two-dimensional topology prediction

The topology of a protein structure provides a brief description of its fold, including only the sequence of secondary structure elements along with their approximate orientations and relative spatial coordinates. This information can be embodied in a two-dimensional (2D) diagram of protein topology. Protter (38) web server was used to generate a pictorial representation of the 2D topology of YXXΦ[I/L/M/F/V] motifs along with YXXΦ-like tetrapeptides in nucleoprotein sequences. Protter predicts transmembrane sites *via* experimental proteomic data as well as annotated sequence information. 2D structure prediction showed the N and C terminal topology of nucleoprotein sequences.

### Prediction of secondary structure elements and internal disordered regions

Predicting the secondary structure of the proteins provide a meaningful insight into the functions of proteins which serve as initial steps toward the tertiary structure prediction. In the current study, DeepGSH (39) web server was used for predicting secondary structural elements including α-helices, coils, and, β-sheets of nucleoprotein sequences within PUUV, SEOV, DOBV, and HTNV. This in turn provides a snapshot of the location of YXXΦ[I/L/M/F/V] motifs, including YXXΦ-like tetrapeptides within viral sequences. DeepGSH showed excellent robustness and better performance than existing tools, thus, DeepGSH was suitable for predicting secondary structural features.

Internal disordered regions exhibit different flavors or types of disorder. PONDR (Predictor of Natural Disordered Regions) (40) was used to calculate the per-residue disorder distribution in nucleoprotein sequences of PUUV, SEOV, DOBV, and HTNV. In PONDR webserver, XL1_XT predictor was selected for predicting disordered regions while other parameters were kept by default. The PONDR web server uncovers long and short disordered regions that might differ in their amino acid characteristics.

### *In silico* prediction and characterization of post-translational modifications

Post-translational modifications (PTMs) can happen at any step of the protein lifespan and have a key role in many different biological processes by influencing dynamics and structure of proteins. By modifying inter- and intramolecular interactions, PTMs not only add new functionalities but also dynamically regulate the activity of proteins. Current study used various tools to predict PTM sites in nucleoprotein sequences of PUUV, SEOV, DOBV, and HTNV. NetPhos-3.1 server (41) was used to prefigure Ser, Tyr, and Thr phosphorylations with default settings. NetPhos-3.1 server used ensembles of neural networks to predict phosphorylation sites in nucleoprotein sequences. Similarly, iNitro-Tyr (42) web server was used for Tyr nitration. Generating a complete and accurate dataset is required to evaluate the Tyr sulfation and its interaction with other Tyr variations. Unfortunately, the experimental identification of sulfation sites is a time- and labor-consuming. As an alternate, computational prediction of sulfation sites using protein primary sequences might provide a helping hand in the identification of putative sulfated residues. Regarding this, GPS-TSP (43) web server was employed to predict the probability of Tyr sulfation within YXXΦ[I/L/M/F/V] motifs.

### Prediction and evaluation of 3D-structure of viral proteins

The three-dimensional (3D) structures of nucleoproteins were predicted using AlphaFold2 (44) to map the positions of both YXXΦ[I/L/M/F/V] motifs as well as YXXΦ-like tetrapeptides of PUUV, SEOV, DOBV, and HTNV in a 3D environment. AlphaFold2 is a neural network-based protein structure prediction program created by Google DeepMind. The AlphaFold2-based prediction was run with the “single sequence” mode. This algorithm first searches for homologous sequences with existing structures to use as a scaffold for placing the new sequence. Additionally, we also specified that the algorithm should run an Amber relaxation procedure to repair any structural violations in the predicted model. AlphaFold2 generated a list of models, but the top-ranked model was selected on the basis of the highest overall pLDDT scores. AlphaFold produces a per-residue estimate of its confidence on a scale from 0 - 100. This confidence measure is called pLDDT and corresponds to the model’s predicted score. Regions with pLDDT > 90 are expected to be modelled to high accuracy. Regions with pLDDT between 70 and 90 are expected to be modelled well (a generally good backbone prediction). While, regions with pLDDT between 50 and 70 are low confidence and should be treated with caution. Lastly, the final predicted structures of PUUV, SEOV, DOBV, and HTNV were superimposed using Chimera X (45) to measure the structural similarity of proteins.

### Immunoreactive epitope prediction analysis

**Figure 1:**
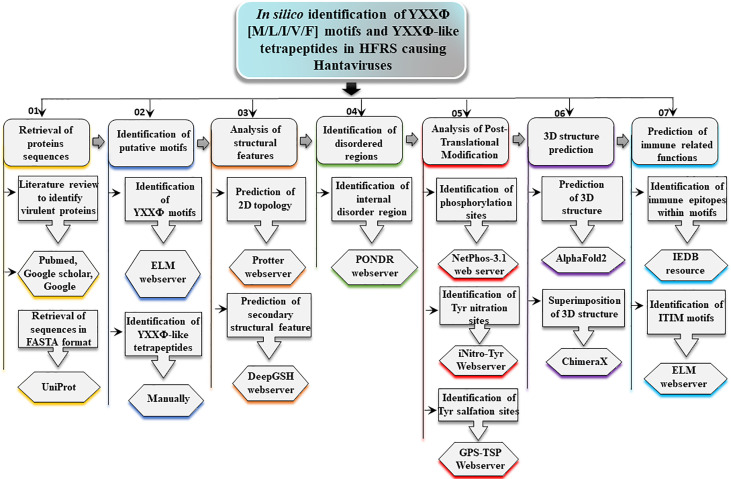
Graphical representation of methodology adopted to uncover YXXΦ[I/L/M/F/V] motifs and YXXΦ-like tetrapeptides within nucleoprotein sequences of PUUV, SEOV, DOBV, and HTNV.

Mapping immune epitopes can provide a helping hand in the identification of antigenic regions within motifs of pathogenic proteins (46, 47). Following that, Immune Epitope Database (IEDB) resource was used to predict immune epitopes within YXXΦ[I/L/M/F/V] motifs and YXXΦ-like tetrapeptides as they might activate B cells by functioning as linear antigenic epitopes (48). In IEDB web server, Chou & Fasman β-turn predictor was used for antibody epitope prediction. Lastly, the Immunoreceptor Tyrosine-based Inhibition Motifs (ITIMs) were predicted using the ELM webserver. ITIM-containing molecules regulate a wide range of biological processes, mainly but not exclusively associated with immunity. The workflow of the present study is displayed in **Figure 1**.

## Results

### Identification of YXXΦ[I/L/M/F/V] motifs and YXXΦ-like tetrapeptides

**Figure 2:**
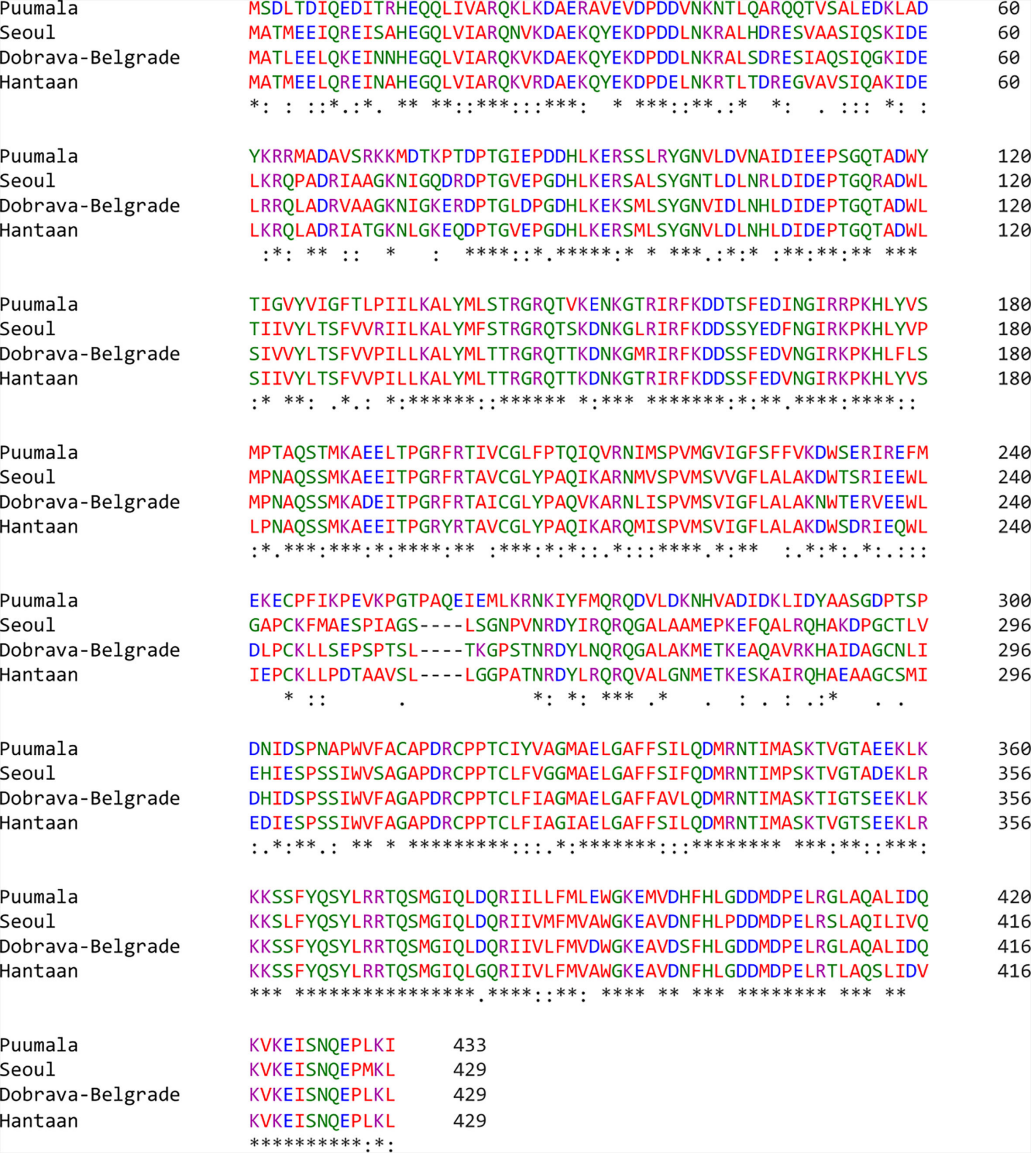
MSA of PUUV, SEOV, DOBV, and HTNV nucleoprotein sequences. Stars: amino acid sequence similarity; colons: amino acid sequence difference.

MSA highlighted a high homology between the nucleoprotein sequence of PUUV, SEOV DOBV, and HTNV (**Figure 2**). HTNV has 82.98% similarity with DOBV, 79.95% similarity with SEOV, and 61.07% similarity with PUUV. These four nucleoprotein sequences were then mapped for potential YXXΦ[I/L/M/F/V] motifs and YXXΦ-like tetrapeptides using ELM web server. It is noteworthy that PUUV, DOBV, and HTNV shared identical YXXΦ[I/L/M/F/V] motif present at position (98–101) and were later named as “Conserved” (YGNV) motif (**Table 1**). Similarly, PUUV has one unique YXXΦ[I/L/M/F/V] motif named as “PUUV-upstream 1”(YVSM) that is located in (178–181) amino acids. SEOV has two unique YXXΦ[I/L/M/F/V] motifs named as “SEOV-upstream 1”(YEDF) and ‘SEOV-upstream 2”(YVPM) motifs which is present at (165–168) and (178–181) amino acids respectively. DOBV has only “Conserved” (YGNV) motif. On the other hand, HTNV has one unique motif named as “HTNV-upstream” which is present at (178–181) amino acids.

**Table 1:** Canonical YXXΦ[I/L/M/F/V] motifs within nucleoprotein sequences of HFRS causing Hantaviruses.

HFRS causing Hantaviruses	Peptide	Position	Motif
PUUV	YGNV	[98]-[101]	Conserved
	YVSM	[178]-[181]	PUUV-upstream
SEOV	YEDF	[165]-[168]	SEOV-upstream 1
	YVPM	[178]-[181]	SEOV-upstream 2
DOBV	YGNV	[98]-[101]	Conserved
HTNV	YGNV	[98]-[101]	Conserved
	YVSL	[178]-[181]	HTNV-upstream

**Figure 3:**
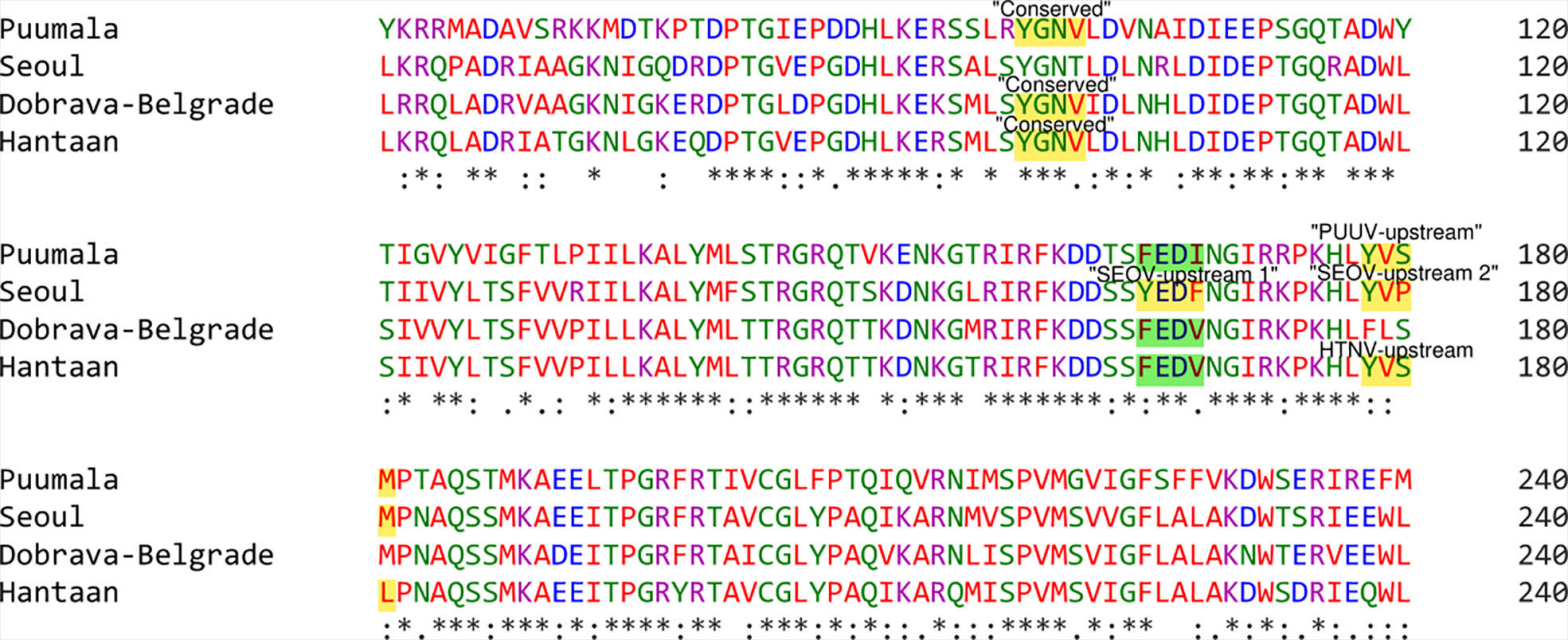
Illustrative representation of YXXΦ[I/L/M/F/V] motifs and YXXΦ-like tetrapeptides within viral sequences. YXXΦ[I/L/M/F/V] motifs are highlighted with yellow color and the YXXΦ-like tetrapeptides are highlighted with green color. Stars, amino acid sequence similarity; colons, amino acid sequence difference.

Following that, some YXXΦ-like tetrapeptides were also predicted in nucleoprotein sequences (**Figure 3**) in which YXXΦ[I/L/M/F/V] motif is “distorted” owing to residual substitutions. In PUUV, DOBV, and HTNV, the “SEOV-upstream 1” has residual substitutions. Therefore, the “SEOV-upstream 1” converted into FEDI (165-168 amino acids) YXXΦ-like tetrapeptides in PUUV, FEDV (165-168 amino acids) YXXΦ-like tetrapeptides in DOBV and HTNV.

### Analysis of structural features

**Figure 4:**
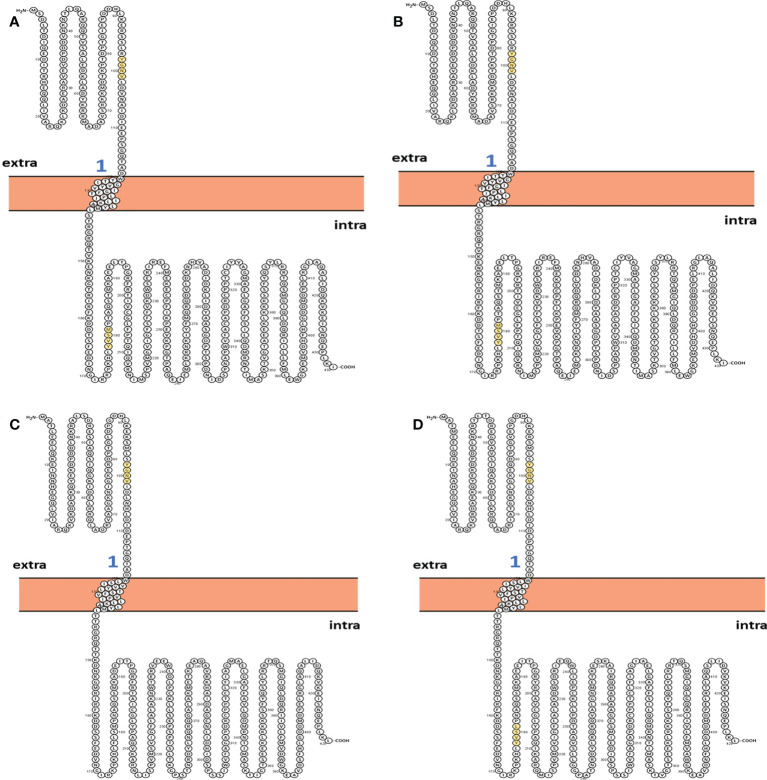
2D Topology of **(A)** PUUV, **(B)** SEOV, **(C)** DOBV, and **(D)** HTNV motifs.

The two-dimensional (2D) topology of motifs and intracellular and extracellular regions were visualized with the Protter web server (**Figure 4**). The 2D topology prediction using Protter demonstrated that nucleoprotein sequences have their C-termini located intracellularly while N termini is extracellularly, confirming Nin-Cout topology. Moreover, the Protter web server predicts that “Conserved” (YGNV) motif is present in extracellular regions while other “PUUV-upstream”, “SEOV-upstream 1”, “SEOV-upstream 2”, and “HTNV-upstream” motifs and YXXΦ-like tetrapeptides present in the intracellular region.

**Figure 5:**
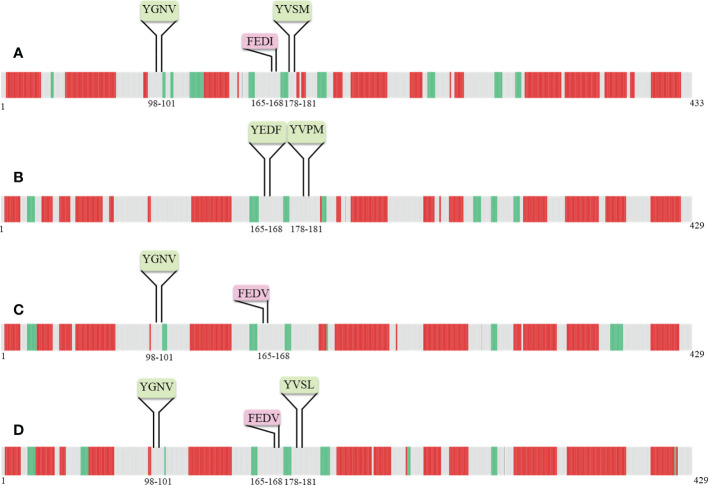
Pictorial representation of secondary structure of **(A)** PUUV, **(B)** SEOV, **(C)** DOBV, and **(D)** HTNV nucleoprotein sequences. α-helices represented with red color, β-strand represented with green color, while grey color indicated coils.

Secondary structure predictions are increasingly becoming the working horse for predicting the function of motifs. Furthermore, the secondary structural elements particularly α-helices, coils, and, β-sheets of nucleoprotein sequences were uncovered with the DeepGSH server (**Figure 5**). DeepGSH server revealed that “Conserved” (YGNV) motif, “PUUV-upstream” (YVSM), “SEOV-upstream 1”(YEDF), and “SEOV-upstream 2”(YVPM), “HTNV-upstream” (YVSL) are situated in coil segments. While YXXΦ-like tetrapeptides were also found to be located on short coil segments. No other differences were observed.

Internal disordered regions in proteins are a class of proteins that do not adopt stable secondary or tertiary structure but instead exist in a heterogeneous ensemble of conformations. Such regions give viral proteins a great flexibility and enable fast adaptability to the host environment, evasion of host defense mechanisms, and survival. Regarding this, PONDR-XL1_XT predictor was employed, revealing the disordered and ordered regions of nucleoprotein sequences. It is worth noting that all YXXΦ-like tetrapeptides and YXXΦ[I/L/M/F/V] motifs are present in the ordered region of viral proteins (**Table 2**).

**Table 2:** Enlists internal disordered regions of PUUV, SEOV, DOBV, and HTNV.

HFRS causing Hantaviruses	Predicted disorder segment	Position	Average Strength
PUUV	EQQLIVARQKLKDAERAVEVDPDDVNKNTLQARQQTVSALEDKL	[15]-[58]	0.8207
	DAVSRKKMDTKPTDPTGIEPDDHLKERSSLR	[67]-[97]	0.8793
	AIDIEEPSG	[106]-[114]	0.6733
	YVIGFTL	[125]-[131]	0.6363
	GRQTVKENKGTRIRFKDDTSFEDING	[145]-[170]	0.7767
	STMKAEEL	[186]-[193]	0.5958
	AQEI	[257]-[260]	0.5554
	NKIYFM	[266]-[271]	0.5624
	PDNI	[300]-[303]	0.5639
	TVG	[351]-[353]	0.5330
	YLRRT	[369]-[373]	0.5582
	PELRGLAQALID	[408]-[419]	0.7283
SEOV	EGQLVIARQNVKDAEKQYEKDPDDLNKRALHDRESVAASIQSKIDELKRQPADRIAAGKNIGQDRDPTGVEPGDHLKERSAL	[15]-[96]	0.8338
	IDEPT	[109]-[113]	0.6265
	YLTSFVVR	[125]-[132]	0.7346
	RGRQTSKDNKGL	[144]-[155]	0.8379
	AQSSMKAEE	[184]-[192]	0.6596
	SLSGNPVNRDYIRQRQGALAAMEPKEFQ	[255]-[282]	0.6733
	PSKTVGTADEK	[344]-[354]	0.6212
	YLRRT	[365]-[369]	0.5539
	QI	[411]-[412]	0.5414
	IV	[414]-[415]	0.5421
DOBV	EGQLVIARQKVKDAEKQYEKDPDDLNKRALSDRESIAQSIQGKIDELRRQLADRVAAGKNIGKERDPTGLDPGDHLKEKS	[15]-[94]	0.8590
	YLTSFVVP	[125]-[132]	0.7142
	RQTTKDNKGMRIRFKDDSSFEDVNG	[146]-[170]	0.8894
	KPKHLFLSMPNAQSSMKADEIT	[173]-[194]	0.6707
	TSLTKGPSTNRDYLNQRQGALAKMETKE	[253]-[280]	0.6627
	SKTIGT	[345]-[350]	0.6475
	YLRRT	[365]-[369]	0.5582
	AVDSFHLGD	[392]-[400]	0.5810
	PELRGLAQALID	[404]-[415]	0.7333
HTNV	EGQLVIARQKVRDAEKQYEKDPDELNKRTLTDREGVAVSIQAKIDELKRQLADRIATGKNLGKEQDPTGVEPGDHLKERSML	[15]-[96]	0.8175
	YLTSFVVP	[125]-[132]	0.7153
	RQTTKDNKGTRIRFKDDSSFEDVNG	[146]-[170]	0.8891
	DYLRQRQVALGNMETKES	[264]-[281]	0.6609
	SKTVGTS	[345]-[351]	0.6713
	QSYLRRTQSMGIQL	[363]-[376]	0.6288
	EL	[405]-[406]	0.5078
	D	[415]-[415]	0.5834

### Analysis and characterization of post-translational modifications

Post-Translational Modifications (PTMs), are among the most crucial mechanisms for enhancing, modifying, or suppressing the functions of proteins. Although analyzing these changes poses difficult problems, its identification yields valuable insights about their active role in biological processes. As PTMs has a key role to play in the regulation of biological processes, therefore these modifications may be conserved across orthologous proteins. At the same time, the divergence of PTMs might contribute to phenotypic diversity. In the framework of the present study, different tools and databases were used to identify PTM sites within both YXXΦ-like tetrapeptides and YXXΦ[I/L/M/F/V] motifs.

**Figure 6:**
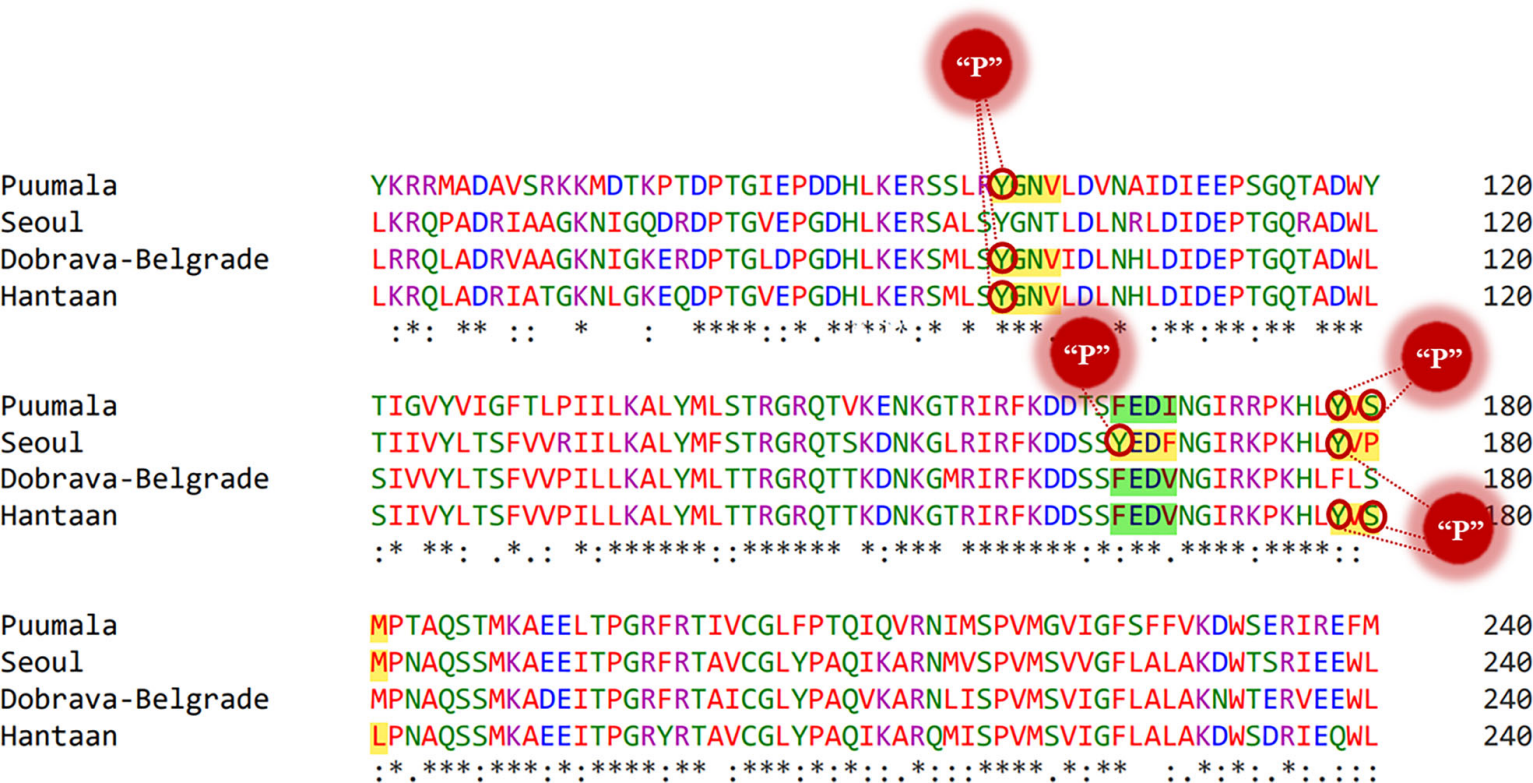
Representation of phosphorylated amino acids within the YXXΦ[I/L/M/F/V] motifs of viral sequences. Stars, amino acid sequence similarity; colons, amino acid sequence difference.

Phosphorylation cascades involve a series of mechanisms for controlling the cell-division cycle, two-component systems, and signal transductions. Prediction of phosphorylated residues within the YXXΦ[I/L/M/F/V] motifs of nucleoproteins using NetPhos3.1 web server uncovered that Tyr98 of YGNV (conserved YXXΦ motif) can be phosphorylated in PUUV, DOB, and HTNV (**Figure 6**). Similarly, Tyr178 and Ser 180 of the “PUUV-upstream motif” (YVSM) can be phosphorylated. In case of SEOV, the Tyr165 and Tyr 178 of the ‘SEOV-upstream 1” motif (YEDF)and SEOV-upstream 2 motif (YVPM) can be phosphorylated. In the same vein, Tyr178 and Ser 180 of “HTNV-upstream” (YVSL) was found to be phosphorylated in the HTNV.

**Figure 7:**
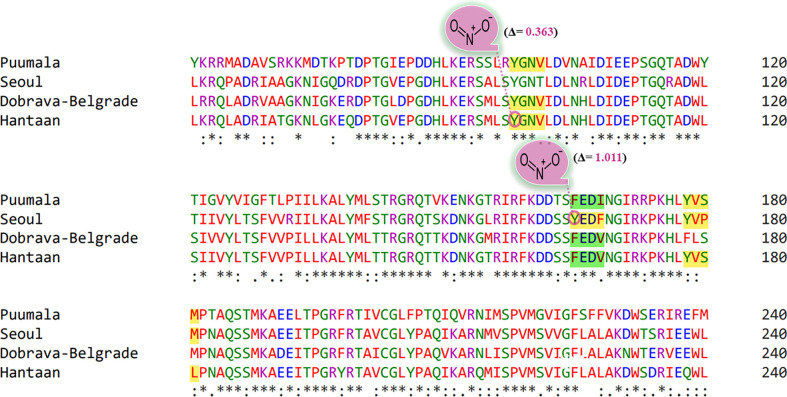
Graphical representation of putative nitrated residues within viral sequences. Stars, amino acid sequence similarity; colons, amino acid sequence difference.

Protein Tyr nitration is a PTM mediated by nitric oxide-derived molecules. Tyr nitration is a free radical [•NO2 (or •NO or ONOO+)] process involving the intermediacy of tyrosyl radicals; in spite of being a nonenzymatic process, nitration is selectively directed toward a limited subset of Tyr residues. Prediction with iNitro-Tyr server revealed that Tyr98 of “Conserved” (YGNV) motif in HTNV is nitrated (**Figure 7**). It is noteworthy that, Leu (at -2 position) increases the probability of Tyr nitration of “Conserved” motif. Similarly, Tyr165 of “SEOV-upstream 1” (YEDF) motif in SEOV is nitrated. No other Tyr was found to be nitrated in PUU and DOBV.

**Figure 8:**
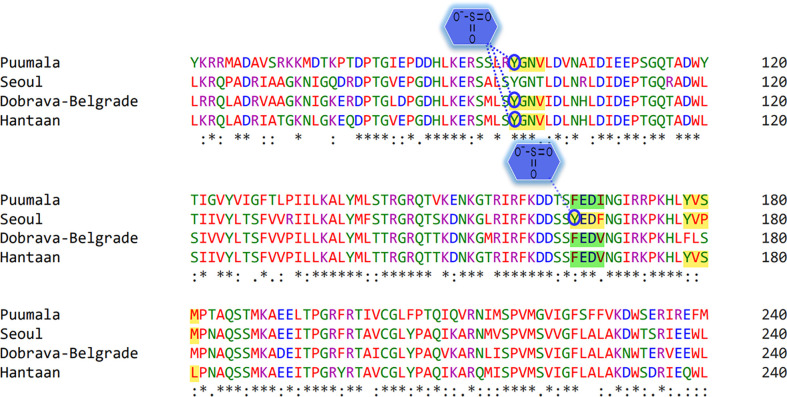
Representation of sulfonated sites within the YXXΦ[I/L/M/F/V] motifs of viral sequences. Stars, amino acid sequence similarity; colons, amino acid sequence difference.

Tyr sulfation is a PTM in which a sulfonate group from the donor 3′-phosphoadenosine 5′-phosphosulfate (PAPS) is transferred to the hydroxyl group of a peptidyltyrosine residue. In this regard, GPS-TSP prediction server uncovered that Tyr98 of “Conserved” (YGNV) motif is sulfonated in PUUV, DOBV, and HTNV (**Figure 8**). In case of SEOV, Tyr165 of “SEOV-upstream 1” (YEDF) motif is also sulfonated. However, no other YXXΦ motifs were found to be sulfonated. In short, PTMs facilitate virus proliferation by accelerating viral assembly and replication during infection. Thus, PTM prediction is a beneficial method to understand viral pathogenesis and replication better.

### Prediction and evaluation of 3D-Structure of viral proteins

**Figure 9:**
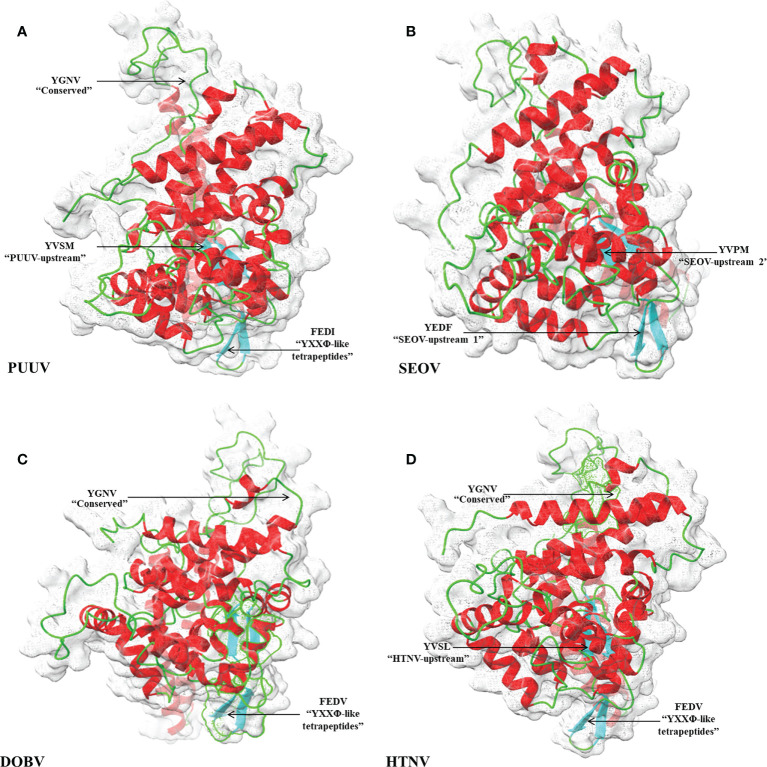
Structural representation of 3D structure **(A)** PUUV **(B)** SEOV **(C)** DOBV **(D)** HTNV. In structure, green represented coils, red represented α-helices and cyanrepresented β-strand. The position of YXXΦ[I/L/M/F/V] motifs and YXXΦ-like tetrapeptides is represented with arrows.

**Figure 10:**
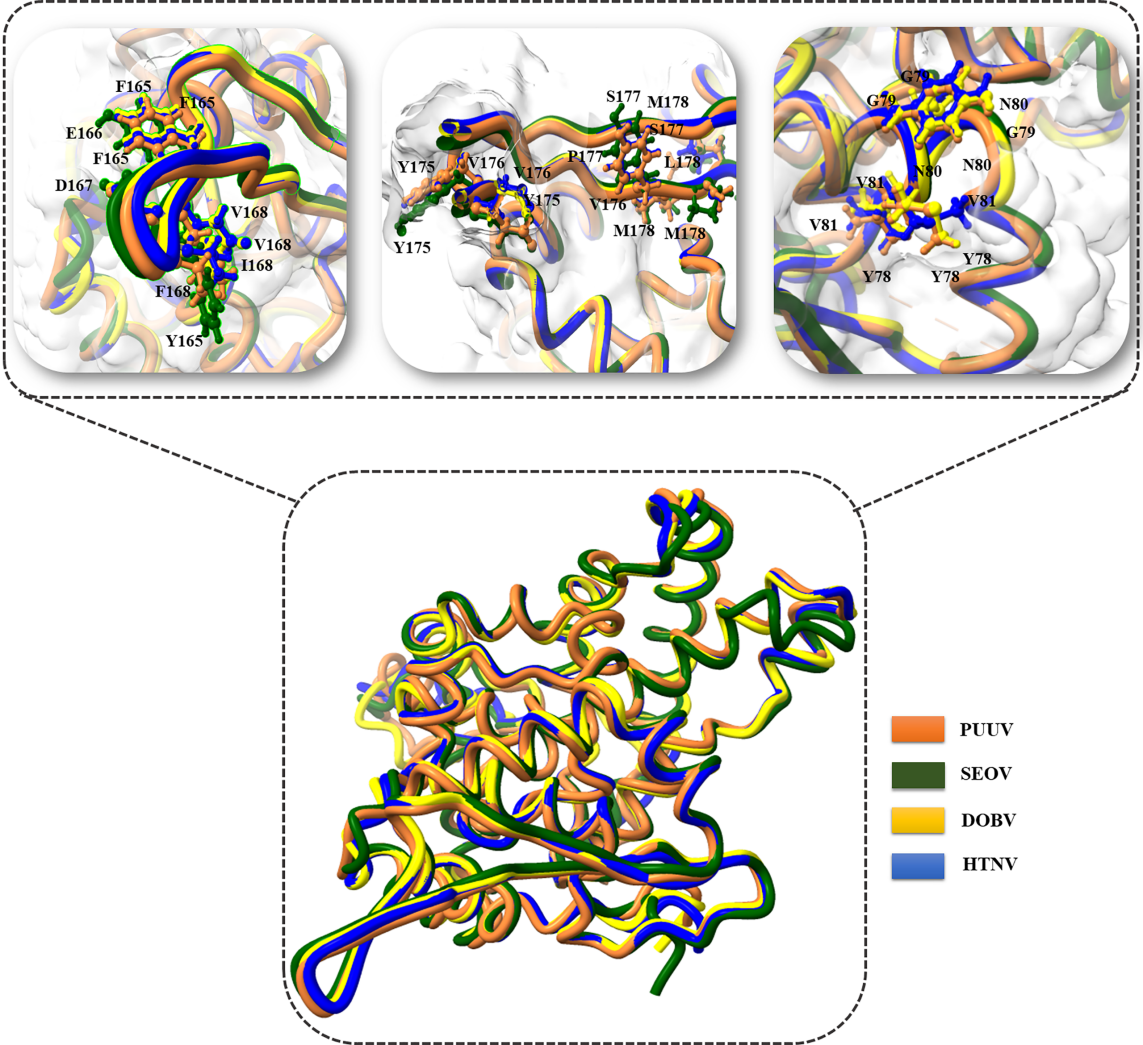
Superimposition of PUUV, SEOV, DOBV, and HTNV.

3D structural models of the nucleoproteins were generated using Alphafold2. The AlphaFold2-based prediction was run with the “mmseqs2” mode by ColabFold. Furthermore, to further improve protein structure quality, we used amber force fields to fix structural violations in the 3D structure of nucleoproteins. Later, AlphaFold2 generated five models for each input sequence. In all five models predicted by AlphaFold2, the top-ranked models were selected based on the pLDDT values. The 3D structures of nucleoproteins provide a comprehensive snapshot of YXXΦ[I/L/M/F/V] motifs and YXXΦ-like tetrapeptides in a 3D environment. **Figure 9** shows the 3D structure of nucleoproteins and the position of motifs and motif-like sequences which is indicated with black arrows. Furthermore, the structure of PUUV, SEOV, DOBV, and HTNV were then superimposed using ChimeraX to unravel the structural homology between motifs of viral sequences (**Figure 10**).

### Immune-related functions of YXXΦ[I/L/M/F/V] motifs and YXXΦ-like tetrapeptides

**Figure 11:**
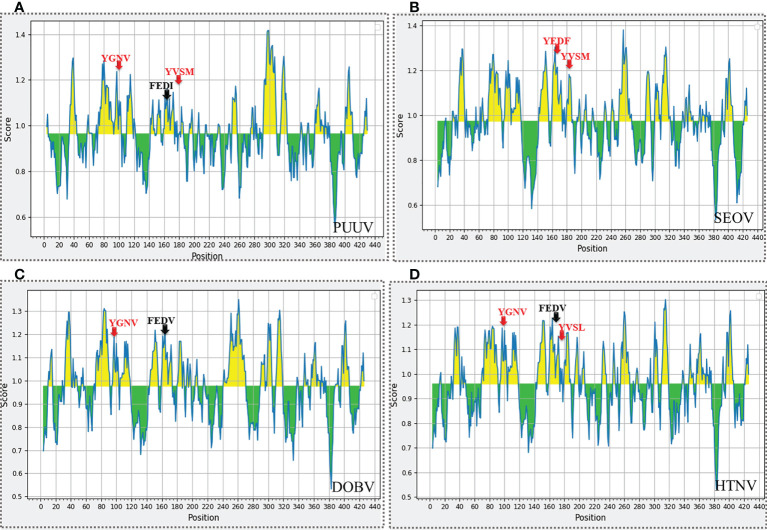
Immune epitope prediction of YXXΦ[I/L/M/F/V] motifs (in red color) and YXXΦ-like tetrapeptides (in black color) *via* Chou & Fasman method of β-turn prediction. **(A)** PUUV, **(B)** SEOV, **(C)** DOBV, and **(D)** HTNV. Those amino acids having scores >0.95 are highlighted with yellow while other amino acids were represented with green.

The nucleoproteins of hantaviruses have immunodominant antigen and these nucleoproteins has conserved antigenicity in contrast to glycoproteins of hantaviruses. Nucleoprotein has thus been employed for serological diagnosis and seroepidemiological research. Consequently, a comprehensive understanding of nucleoproteins is mandatory to stop the process of replication in hantavirus (49). Regarding this, the immune epitopes of YXXΦ-like tetrapeptides and YXXΦ[I/L/M/F/V] motifs were predicted using Chou & Fasman β-turn predictor available in IEDB server. The Chou & Fasman β-turn predictor evaluates the antigenic properties on the basis of β-turn structure scale. It implies that the epitope has always been turned in a β shape (50). The analysis with Chou & Fasman β-turn predictor revealed that, predicted motifs and motif-like tetrapeptides belong to antigenic regions of nucleoproteins (**Figure 11**; **Supplementary Table S1**). Those who met the precise criteria of average score >0.95 were considered epitopes (**Table 3**).

**Table 3:** Enlist immune epitopes within YXXΦ[I/L/M/F/V] motifs.

Motif	Residue	Start	End	Sequence	Average score
PUUV					
YGNV	Y	95	101	SLRYGNV	1.104
	G	96	102	LRYGNVL	0.984
	N	97	103	RYGNVLD	1.109
	V	98	104	YGNVLDV	1.044
YVSM	Y	175	181	KHLYVSM	0.889
	V	176	182	HLYVSMP	0.961
	S	177	183	LYVSMPT	0.963
	M	178	184	YVSMPTA	0.973
SEOV					
YEDF	Y	162	168	DSSYEDF	1.18
	E	163	169	SSYEDFN	1.194
	D	164	170	SYEDFNG	1.213
	F	165	171	YEDFNGI	1.076
YVPM	Y	175	181	KHLYVPM	0.901
	V	176	182	HLYVPMP	0.974
	P	177	183	LYVPMPN	1.061
	M	178	184	YVPMPNA	1.071
DOBV					
YGNV	Y	95	101	MLSYGNV	1.054
	G	96	102	LSYGNVI	1.036
	N	97	103	SYGNVID	1.16
	V	98	104	YGNVIDL	1.04
HTNV					
YGNV	Y	95	101	MLSYGNV	1.054
	G	96	102	LSYGNVL	1.053
	N	97	103	SYGNVLD	1.177
	V	98	104	YGNVLDL	1.057
YVSL	Y	175	181	KHLYVSL	0.887
	V	176	182	HLYVSLP	0.96
	S	177	183	LYVSLPN	1.047
	L	178	184	YVSLPNA	1.057

**Figure 12:**
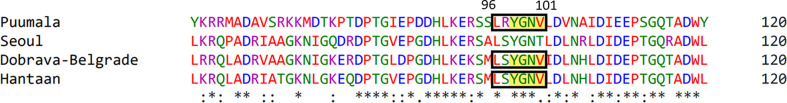
Representation of ITIM motif within YXXΦ[I/L/M/F/V] motifs.

The nucleoprotein is predicted to contain multiple signaling motifs predicted by the ELM web server, including immunoreceptor tyrosine-based inhibitory motifs (ITIM). Finally, we searched whether YXXΦ[I/L/M/F/V] motifs or YXXΦ-like tetrapeptides of nucleoproteins could be an element of an ITIM motif, as recent studies provide strong shreds of evidences that ITIM motifs are responsible for the regulation and evasion of host immune responses in viruses (51). Intriguingly, nucleoprotein analysis using ELM server revealed that the “Conserved” (YGNV) motif belongs to ITIM (LRYGNV) motif located at position (96–101) amino acids (**Figure 12**).

## Discussion

HFRS is a potentially fatal infectious disease with worldwide distribution. PUUV, SEOV, DOBV, and HTNV are the primary causative agents of HFRS (52). PUUV, SEOV, DOBV, and HTNV are a member of rodent-borne viruses called hantaviruses that cause life-threatening human diseases in Europe and Asia. The enveloped and single-stranded RNA genome of hantaviruses is tri-segmented into small, medium, and large (S, M and L) segments. The hantavirus nucleocapsid protein comprises 429 to 433 amino acids (about 50 kDa in size) (21, 53). It has been shown that the early immune response in people with hantavirus infections are mostly targeted toward the nucleoproteins. The nucleoproteins are highly conserved in the hantavirus genus and are one of the most abundant structural proteins in virus-infected cells (54, 55). Evidence suggests that substantial amounts of nucleoproteins are expressed early after infection. Nucleoproteins express in the cytoplasm of infected cell (56). Hantavirus nucleoprotein controls viral replication and assembly, encapsidates viral RNA, and plays a crucial part in the virus life cycle (57). Consequently, various diagnostics methods have now been developed based on recognizing the hantaviruses nucleoprotein or an anti-nucleoprotein antibody to halt the pathogenesis of viral infection at early stages (58, 59).

Fever and flu-like symptoms are the hallmarks of HFRS, which evolve into anemia, acute kidney damage with severe thrombocytopenia and coagulation disorders (60). Supportive therapy is the mainstay of care for patients with hantavirus infections. Care includes careful management of the patient’s fluid (hydration) and electrolyte (e.g., sodium, potassium, chloride) levels, maintenance of correct oxygen and blood pressure levels, and appropriate treatment of any secondary infections. There is currently no effective treatment available for either HFRS. Recently, Neveu et al. (61) identify YXXΦ motif within core proteins of hepatitis C virus and demonstrated that YXXΦ motif plays significant role in viral assembly. Further, Kakkanas et al. (29) used the same methodology and identified YXXΦ Motifs of the SARS Coronaviruses 1 and 2 ORF3a Peptides and proposed that targeting these YXXΦ Motifs might help in the prediction of by novel Host-virus interactions. This study revolves around the identification of tyrosine-based motif (YXXΦ[I/L/M/F/V]) and YXXΦ-like tetrapeptides within nucleoprotein sequences of PUUV, SEOV, DOBV, and HTNV to identify novel anti-viral targets. YXXΦ[I/L/M/F/V] motifs have key role to play in regulating immune responses. At first, homology between nucleoproteins of PUUV, SEOV, DOBV, and HTNV were determined which revealed high homology among these hantaviruses. Despite that, the protein sequences of PUUV, SEOV, DOBV, and HTNV shows several differences which highlighted that residual substitution generated YXXΦ-like tetrapeptides and these YXXΦ-like tetrapeptides are later prone to certain PTMs which ultimately affect the proper functioning of nucleoproteins. The other factor like disordered regions near YXXΦ[I/L/M/F/V] motifs and YXXΦ-like tetrapeptides also affect the functionalities of these motifs.

Different PTMs sites were then observed within YXXΦ[I/L/M/F/V] motifs as well as YXXΦ-like tetrapeptides. Various biological and biomedical processes are governed mainly by protein phosphorylation. Esterification of residue by the addition of phosphate causes conformational changes in the protein, ultimately leading to protein activity or stability variations. Proteins can be phosphorylated on Ser, Thr, or Tyr residues. This PTM has the potential to alter the enzymatic activity, subcellular location, and stability of proteins with diverse roles in cells. Our study proposed phosphorylation of some Tyr and Ser residues within YXXΦ[I/L/M/F/V] motifs. In the light of recent literature, phosphorylation of Tyr within tyrosine-based motifs induce hinders the endocytosis of protein containing that particular motif (62, 63). While the phosphorylation of Ser influences the sorting of protein (64). If we compare our findings with published literature then it is worth noting that phosphorylation of Tyr and Ser residues within YXXΦ[I/L/M/F/V] motifs of PUUV, SEOV, DOBV, and HTNV hinders the endocytosis and affect the sorting process of nucleoproteins.

Tyr sulfation is expected to happen almost exclusively on secreted proteins and transmembrane-spanning proteins. According to available data, an organism’s total protein content can contain up to 1% sulfated Tyr residues. Therefore, the most frequent PTM on Tyr residues is Tyr sulfation (65, 66). Sulfated Tyr residues are currently engaged in several biological processes, mainly related to ligand binding and host–viral interactions (67). Our study predicts that sulfonated Tyr residues in YXXΦ[I/L/M/F/V] motifs. Considering that sulfation increases a protein’s molecular weight like phosphorylation, thus making the altered residues visible on the cell surface (68), it makes sense to assume that the nucleoprotein may engage in previously unrecognized interactions with extracellular host proteins upon sulfation.

Protein Tyr nitration is a covalent and specific modification of Tyr residues resulting in alteration of structure and function of the protein. It is a free radical process involving the reaction of a tyrosyl radical with •NO to form 3-NO-Tyr followed by sequential 2-electron oxidation to 3-NO-Tyr through Tyr iminoxyl radical. Importantly, not all Tyr residues within a protein sequence are amenable to nitration, therefore, Tyr nitration is not a random process, in fact, it is considered very specific. Recent studies reported that nitration of Tyr completely alters the function of protein which ultimately lead to viral pathogenesis (69). Thus, the nitration of Tyr residues in nucleoproteins of PUUV, SEOV, DOBV, and HTNV could significantly affect viral pathogenesis and survival.

Later, 3D structures of PUUV, SEOV, DOBV, and HTNV nucleoproteins were predicted using AlphaFold2 to map the location of YXXΦ [I/L/M/F/V] motifs and YXXΦ-like tetrapeptides in 3D environment. Finally, the Chou & Fasman β-turn predictor revealed that proposed motifs have good antigenic properties. Lastly, “Conserved” (YGNV) motif at position (96–101) amino acids found to be part of ITIM Tyr-based Immunoreceptor motif (LRYGNV) motif. This study also has some limitations as without any *in vitro* and *in vivo* validation, the proposed YXXΦ[I/L/M/F/V] motifs and YXXΦ-like tetrapeptides can be questioned very easily because the predictive power of algorithms used in tools is relatively low. Fascinatingly, the ones that exist might instruct the tools to produce more accurate data. However, conformational research on the effect of PTMs on the functionality of proposed motifs is urgently needed to better understand their role in viral pathogenesis, virulence, and replication. This ultimately opens up new and exciting avenues for the treatment of HFRS.

To sum up, our work explored the differences among nucleoprotein sequences of PUUV, SEOV, DOBV, and HTNV, especially within YXXΦ[I/L/M/F/V] motifs and YXXΦ-like tetrapeptides. Overall, these studies highlight region of nucleoproteins that play critical roles in the biological function and where changes on a single residue have dramatic effects on virus replicative capacity. Furthermore, we envision that the targeting of the YXXΦ motifs with pharmacological reagents that will induce PTMs with suitable deleterious effects to the structural integrity and the immunological properties of nucleoproteins, could assist with the rational design of anti-HFRS treatments. We strongly believe that combining anti-HFRS drug with YXXΦ[I/L/M/F/V] motifs and YXXΦ-like tetrapeptides may offer synergistic effects which ultimately produce fruitful outcome in controlling the viral infection worldwide.

## Conclusion

HFRS is acute interstitial nephropathy characterized by high fever and varying degrees of renal insufficiency and hemorrhage. The nucleocapsid protein of hantaviruses represents an impressive example of a viral multifunctional protein. Despite that, nucleoproteins of PUUV, SEOV, DOBV, and HTNV are approximately similar in length; however, they exhibit considerable differences in their N-termini and residual substitutions throughout the length of nucleoprotein sequences, particularly within YXXΦ[I/L/M/F/V] motifs and YXXΦ-like tetrapeptides. The predicted 3D models and PTMs can attribute diverse functions to the nucleoproteins of PUUV, SEOV, DOBV, and HTNV. Thus, discovering such mechanisms can provide novel targets to develop potential antiviral drugs for the treatment of HFRS. Additional experimental research on this putative YXXΦ [I/L/M/F/V] motifs and YXXΦ-like tetrapeptides lead to an increase in our knowledge to fight against HFRS in the future by employing novel therapeutic approaches. Although it is difficult to draw conclusions about the biological functions of a protein from bioinformatically predicted structures, we hope that the results of our work will boost *in vitro* experimentation on this fascinating biomolecule, nucleoprotein that will ultimately assign new biological roles to it.

## Data availability statement

The datasets presented in this study can be found in online repositories. The names of the repository/repositories and accession number(s) can be found in the article/**Supplementary Material**.

## Author contributions

FN drafted the manuscript, prepared illustrations, and discussed the content with the other authors. UA conceived the topic and revised the content of the manuscript. MAs and MM revised the manuscript. AA and MAl were involved in the final development of the project and funding acquisition. All authors contributed to the article and approved the submitted version.

## Funding

Authors are thankful to the Researchers Supporting Project number (RSP2022R491), King Saud University, Riyadh, Saudi Arabia.

## Conflict of interest

The authors declare that the research was conducted in the absence of any commercial or financial relationships that could be construed as a potential conflict of interest.

## Publisher’s note

All claims expressed in this article are solely those of the authors and do not necessarily represent those of their affiliated organizations, or those of the publisher, the editors and the reviewers. Any product that may be evaluated in this article, or claim that may be made by its manufacturer, is not guaranteed or endorsed by the publisher.

## Supplementary material

Supplementary Table 1 Linear antigenic epitopes of nucleoproteins.
