# Supplementary material for: Comprehensive computational analysis reveals YXXΦ[I/L/M/F/V] motif and YXXΦ-like tetrapeptides across HFRS causing Hantaviruses and their association with viral pathogenesis and host immune regulation
Source: Front Immunol. 2022 Oct 6;13:1031608. doi: 10.3389/fimmu.2022.1031608 (PMC9584616; doi:10.3389/fimmu.2022.1031608)
Supplement: Supplementary Table 1 — Linear antigenic epitopes of nucleoproteins. [file Table_1.docx]

**Supplementary Table S1.** Linear antigenic epitopes of nucleoproteins

| **Puumala virus** | | | | | |
| --- | --- | --- | --- | --- | --- |
| **Position** | **Residue** | **Start** | **End** | **Peptide** | **Score** |
| 4 | L | 1 | 7 | MSDLTDI | 0.996 |
| 5 | T | 2 | 8 | SDLTDIQ | 1.05 |
| 6 | D | 3 | 9 | DLTDIQE | 0.951 |
| 7 | I | 4 | 10 | LTDIQED | 0.951 |
| 8 | Q | 5 | 11 | TDIQEDI | 0.934 |
| 9 | E | 6 | 12 | DIQEDIT | 0.934 |
| 10 | D | 7 | 13 | IQEDITR | 0.861 |
| 11 | I | 8 | 14 | QEDITRH | 0.93 |
| 12 | T | 9 | 15 | EDITRHE | 0.896 |
| 13 | R | 10 | 16 | DITRHEQ | 0.93 |
| 14 | H | 11 | 17 | ITRHEQQ | 0.861 |
| 15 | E | 12 | 18 | TRHEQQL | 0.879 |
| 16 | Q | 13 | 19 | RHEQQLI | 0.809 |
| 17 | Q | 14 | 20 | HEQQLIV | 0.744 |
| 18 | L | 15 | 21 | EQQLIVA | 0.703 |
| 19 | I | 16 | 22 | QQLIVAR | 0.733 |
| 20 | V | 17 | 23 | QLIVARQ | 0.733 |
| 21 | A | 18 | 24 | LIVARQK | 0.737 |
| 22 | R | 19 | 25 | IVARQKL | 0.737 |
| 23 | Q | 20 | 26 | VARQKLK | 0.814 |
| 24 | K | 21 | 27 | ARQKLKD | 0.951 |
| 25 | L | 22 | 28 | RQKLKDA | 0.951 |
| 26 | K | 23 | 29 | QKLKDAE | 0.921 |
| 27 | D | 24 | 30 | KLKDAER | 0.917 |
| 28 | A | 25 | 31 | LKDAERA | 0.867 |
| 29 | E | 26 | 32 | KDAERAV | 0.854 |
| 30 | R | 27 | 33 | DAERAVE | 0.816 |
| 31 | A | 28 | 34 | AERAVEV | 0.679 |
| 32 | V | 29 | 35 | ERAVEVD | 0.793 |
| 33 | E | 30 | 36 | RAVEVDP | 0.904 |
| 34 | V | 31 | 37 | AVEVDPD | 0.977 |
| 35 | D | 32 | 38 | VEVDPDD | 1.091 |
| 36 | P | 33 | 39 | EVDPDDV | 1.091 |
| 37 | D | 34 | 40 | VDPDDVN | 1.209 |
| 38 | D | 35 | 41 | DPDDVNK | 1.281 |
| 39 | V | 36 | 42 | PDDVNKN | 1.296 |
| 40 | N | 37 | 43 | DDVNKNT | 1.216 |
| 41 | K | 38 | 44 | DVNKNTL | 1.091 |
| 42 | N | 39 | 45 | VNKNTLQ | 1.023 |
| 43 | T | 40 | 46 | NKNTLQA | 1.046 |
| 44 | L | 41 | 47 | KNTLQAR | 0.959 |
| 45 | Q | 42 | 48 | NTLQARQ | 0.954 |
| 46 | A | 43 | 49 | TLQARQQ | 0.871 |
| 47 | R | 44 | 50 | LQARQQT | 0.871 |
| 48 | Q | 45 | 51 | QARQQTV | 0.859 |
| 49 | Q | 46 | 52 | ARQQTVS | 0.923 |
| 50 | T | 47 | 53 | RQQTVSA | 0.923 |
| 51 | V | 48 | 54 | QQTVSAL | 0.871 |
| 52 | S | 49 | 55 | QTVSALE | 0.837 |
| 53 | A | 50 | 56 | TVSALED | 0.906 |
| 54 | L | 51 | 57 | VSALEDK | 0.913 |
| 55 | E | 52 | 58 | SALEDKL | 0.926 |
| 56 | D | 53 | 59 | ALEDKLA | 0.816 |
| 57 | K | 54 | 60 | LEDKLAD | 0.93 |
| 58 | L | 55 | 61 | EDKLADY | 1.009 |
| 59 | A | 56 | 62 | DKLADYK | 1.047 |
| 60 | D | 57 | 63 | KLADYKR | 0.974 |
| 61 | Y | 58 | 64 | LADYKRR | 0.966 |
| 62 | K | 59 | 65 | ADYKRRM | 0.967 |
| 63 | R | 60 | 66 | DYKRRMA | 0.967 |
| 64 | R | 61 | 67 | YKRRMAD | 0.967 |
| 65 | M | 62 | 68 | KRRMADA | 0.899 |
| 66 | A | 63 | 69 | RRMADAV | 0.826 |
| 67 | D | 64 | 70 | RMADAVS | 0.894 |
| 68 | A | 65 | 71 | MADAVSR | 0.894 |
| 69 | V | 66 | 72 | ADAVSRK | 0.953 |
| 70 | S | 67 | 73 | DAVSRKK | 1.003 |
| 71 | R | 68 | 74 | AVSRKKM | 0.88 |
| 72 | K | 69 | 75 | VSRKKMD | 0.994 |
| 73 | K | 70 | 76 | SRKKMDT | 1.06 |
| 74 | M | 71 | 77 | RKKMDTK | 1 |
| 75 | D | 72 | 78 | KKMDTKP | 1.081 |
| 76 | T | 73 | 79 | KMDTKPT | 1.074 |
| 77 | K | 74 | 80 | MDTKPTD | 1.139 |
| 78 | P | 75 | 81 | DTKPTDP | 1.27 |
| 79 | T | 76 | 82 | TKPTDPT | 1.199 |
| 80 | D | 77 | 83 | KPTDPTG | 1.284 |
| 81 | P | 78 | 84 | PTDPTGI | 1.207 |
| 82 | T | 79 | 85 | TDPTGIE | 1.096 |
| 83 | G | 80 | 86 | DPTGIEP | 1.176 |
| 84 | I | 81 | 87 | PTGIEPD | 1.176 |
| 85 | E | 82 | 88 | TGIEPDD | 1.167 |
| 86 | P | 83 | 89 | GIEPDDH | 1.166 |
| 87 | D | 84 | 90 | IEPDDHL | 1.027 |
| 88 | D | 85 | 91 | EPDDHLK | 1.104 |
| 89 | H | 86 | 92 | PDDHLKE | 1.104 |
| 90 | L | 87 | 93 | DDHLKER | 1.023 |
| 91 | K | 88 | 94 | DHLKERS | 1.019 |
| 92 | E | 89 | 95 | HLKERSS | 1.014 |
| 93 | R | 90 | 96 | LKERSSL | 0.963 |
| 94 | S | 91 | 97 | KERSSLR | 1.014 |
| 95 | S | 92 | 98 | ERSSLRY | 1.033 |
| 96 | L | 93 | 99 | RSSLRYG | 1.15 |
| 97 | R | 94 | 100 | SSLRYGN | 1.237 |
| 98 | Y | 95 | 101 | SLRYGNV | 1.104 |
| 99 | G | 96 | 102 | LRYGNVL | 0.984 |
| 100 | N | 97 | 103 | RYGNVLD | 1.109 |
| 101 | V | 98 | 104 | YGNVLDV | 1.044 |
| 102 | L | 99 | 105 | GNVLDVN | 1.104 |
| 103 | D | 100 | 106 | NVLDVNA | 0.976 |
| 104 | V | 101 | 107 | VLDVNAI | 0.82 |
| 105 | N | 102 | 108 | LDVNAID | 0.957 |
| 106 | A | 103 | 109 | DVNAIDI | 0.94 |
| 107 | I | 104 | 110 | VNAIDIE | 0.837 |
| 108 | D | 105 | 111 | NAIDIEE | 0.871 |
| 109 | I | 106 | 112 | AIDIEEP | 0.866 |
| 110 | E | 107 | 113 | IDIEEPS | 0.976 |
| 111 | E | 108 | 114 | DIEEPSG | 1.131 |
| 112 | P | 109 | 115 | IEEPSGQ | 1.063 |
| 113 | S | 110 | 116 | EEPSGQT | 1.133 |
| 114 | G | 111 | 117 | EPSGQTA | 1.121 |
| 115 | Q | 112 | 118 | PSGQTAD | 1.224 |
| 116 | T | 113 | 119 | SGQTADW | 1.144 |
| 117 | A | 114 | 120 | GQTADWY | 1.103 |
| 118 | D | 115 | 121 | QTADWYT | 1.017 |
| 119 | W | 116 | 122 | TADWYTI | 0.944 |
| 120 | Y | 117 | 123 | ADWYTIG | 1.03 |
| 121 | T | 118 | 124 | DWYTIGV | 1.007 |
| 122 | I | 119 | 125 | WYTIGVY | 0.961 |
| 123 | G | 120 | 126 | YTIGVYV | 0.896 |
| 124 | V | 121 | 127 | TIGVYVI | 0.8 |
| 125 | Y | 122 | 128 | IGVYVIG | 0.886 |
| 126 | V | 123 | 129 | GVYVIGF | 0.904 |
| 127 | I | 124 | 130 | VYVIGFT | 0.819 |
| 128 | G | 125 | 131 | YVIGFTL | 0.831 |
| 129 | F | 126 | 132 | VIGFTLP | 0.886 |
| 130 | T | 127 | 133 | IGFTLPI | 0.881 |
| 131 | L | 128 | 134 | GFTLPII | 0.881 |
| 132 | P | 129 | 135 | FTLPIIL | 0.743 |
| 133 | I | 130 | 136 | TLPIILK | 0.801 |
| 134 | I | 131 | 137 | LPIILKA | 0.759 |
| 135 | L | 132 | 138 | PIILKAL | 0.759 |
| 136 | K | 133 | 139 | IILKALY | 0.704 |
| 137 | A | 134 | 140 | ILKALYM | 0.723 |
| 138 | L | 135 | 141 | LKALYML | 0.74 |
| 139 | Y | 136 | 142 | KALYMLS | 0.86 |
| 140 | M | 137 | 143 | ALYMLST | 0.853 |
| 141 | L | 138 | 144 | LYMLSTR | 0.894 |
| 142 | S | 139 | 145 | YMLSTRG | 1.033 |
| 143 | T | 140 | 146 | MLSTRGR | 1.006 |
| 144 | R | 141 | 147 | LSTRGRQ | 1.06 |
| 145 | G | 142 | 148 | STRGRQT | 1.113 |
| 146 | R | 143 | 149 | TRGRQTV | 0.98 |
| 147 | Q | 144 | 150 | RGRQTVK | 0.987 |
| 148 | T | 145 | 151 | GRQTVKE | 0.957 |
| 149 | V | 146 | 152 | RQTVKEN | 0.957 |
| 150 | K | 147 | 153 | QTVKENK | 0.966 |
| 151 | E | 148 | 154 | TVKENKG | 1.049 |
| 152 | N | 149 | 155 | VKENKGT | 1.049 |
| 153 | K | 150 | 156 | KENKGTR | 1.113 |
| 154 | G | 151 | 157 | ENKGTRI | 1.036 |
| 155 | T | 152 | 158 | NKGTRIR | 1.066 |
| 156 | R | 153 | 159 | KGTRIRF | 0.929 |
| 157 | I | 154 | 160 | GTRIRFK | 0.929 |
| 158 | R | 155 | 161 | TRIRFKD | 0.914 |
| 159 | F | 156 | 162 | RIRFKDD | 0.986 |
| 160 | K | 157 | 163 | IRFKDDT | 0.987 |
| 161 | D | 158 | 164 | RFKDDTS | 1.124 |
| 162 | D | 159 | 165 | FKDDTSF | 1.074 |
| 163 | T | 160 | 166 | KDDTSFE | 1.094 |
| 164 | S | 161 | 167 | DDTSFED | 1.159 |
| 165 | F | 162 | 168 | DTSFEDI | 1.017 |
| 166 | E | 163 | 169 | TSFEDIN | 1.031 |
| 167 | D | 164 | 170 | SFEDING | 1.117 |
| 168 | I | 165 | 171 | FEDINGI | 0.98 |
| 169 | N | 166 | 172 | EDINGIR | 1.03 |
| 170 | G | 167 | 173 | DINGIRR | 1.06 |
| 171 | I | 168 | 174 | INGIRRP | 1.069 |
| 172 | R | 169 | 175 | NGIRRPK | 1.146 |
| 173 | R | 170 | 176 | GIRRPKH | 1.059 |
| 174 | P | 171 | 177 | IRRPKHL | 0.92 |
| 175 | K | 172 | 178 | RRPKHLY | 1.016 |
| 176 | H | 173 | 179 | RPKHLYV | 0.951 |
| 177 | L | 174 | 180 | PKHLYVS | 1.02 |
| 178 | Y | 175 | 181 | KHLYVSM | 0.889 |
| 179 | V | 176 | 182 | HLYVSMP | 0.961 |
| 180 | S | 177 | 183 | LYVSMPT | 0.963 |
| 181 | M | 178 | 184 | YVSMPTA | 0.973 |
| 182 | P | 179 | 185 | VSMPTAQ | 0.95 |
| 183 | T | 180 | 186 | SMPTAQS | 1.083 |
| 184 | A | 181 | 187 | MPTAQST | 1.016 |
| 185 | Q | 182 | 188 | PTAQSTM | 1.016 |
| 186 | S | 183 | 189 | TAQSTMK | 0.943 |
| 187 | T | 184 | 190 | AQSTMKA | 0.9 |
| 188 | M | 185 | 191 | QSTMKAE | 0.911 |
| 189 | K | 186 | 192 | STMKAEE | 0.877 |
| 190 | A | 187 | 193 | TMKAEEL | 0.757 |
| 191 | E | 188 | 194 | MKAEELT | 0.757 |
| 192 | E | 189 | 195 | KAEELTP | 0.889 |
| 193 | L | 190 | 196 | AEELTPG | 0.967 |
| 194 | T | 191 | 197 | EELTPGR | 1.009 |
| 195 | P | 192 | 198 | ELTPGRF | 0.989 |
| 196 | G | 193 | 199 | LTPGRFR | 1.019 |
| 197 | R | 194 | 200 | TPGRFRT | 1.071 |
| 198 | F | 195 | 201 | PGRFRTI | 1.001 |
| 199 | R | 196 | 202 | GRFRTIV | 0.856 |
| 200 | T | 197 | 203 | RFRTIVC | 0.803 |
| 201 | I | 198 | 204 | FRTIVCG | 0.89 |
| 202 | V | 199 | 205 | RTIVCGL | 0.889 |
| 203 | C | 200 | 206 | TIVCGLF | 0.839 |
| 204 | G | 201 | 207 | IVCGLFP | 0.919 |
| 205 | L | 202 | 208 | VCGLFPT | 0.989 |
| 206 | F | 203 | 209 | CGLFPTQ | 1.057 |
| 207 | P | 204 | 210 | GLFPTQI | 0.954 |
| 208 | T | 205 | 211 | LFPTQIQ | 0.871 |
| 209 | Q | 206 | 212 | FPTQIQV | 0.859 |
| 210 | I | 207 | 213 | PTQIQVR | 0.909 |
| 211 | Q | 208 | 214 | TQIQVRN | 0.914 |
| 212 | V | 209 | 215 | QIQVRNI | 0.844 |
| 213 | R | 210 | 216 | IQVRNIM | 0.79 |
| 214 | N | 211 | 217 | QVRNIMS | 0.927 |
| 215 | I | 212 | 218 | VRNIMSP | 1.004 |
| 216 | M | 213 | 219 | RNIMSPV | 1.004 |
| 217 | S | 214 | 220 | NIMSPVM | 0.954 |
| 218 | P | 215 | 221 | IMSPVMG | 0.954 |
| 219 | V | 216 | 222 | MSPVMGV | 0.959 |
| 220 | M | 217 | 223 | SPVMGVI | 0.94 |
| 221 | G | 218 | 224 | PVMGVIG | 0.959 |
| 222 | V | 219 | 225 | VMGVIGF | 0.827 |
| 223 | I | 220 | 226 | MGVIGFS | 0.96 |
| 224 | G | 221 | 227 | GVIGFSF | 0.96 |
| 225 | F | 222 | 228 | VIGFSFF | 0.823 |
| 226 | S | 223 | 229 | IGFSFFV | 0.823 |
| 227 | F | 224 | 230 | GFSFFVK | 0.9 |
| 228 | F | 225 | 231 | FSFFVKD | 0.886 |
| 229 | V | 226 | 232 | SFFVKDW | 0.937 |
| 230 | K | 227 | 233 | FFVKDWS | 0.937 |
| 231 | D | 228 | 234 | FVKDWSE | 0.957 |
| 232 | W | 229 | 235 | VKDWSER | 1.007 |
| 233 | S | 230 | 236 | KDWSERI | 1.003 |
| 234 | E | 231 | 237 | DWSERIR | 0.994 |
| 235 | R | 232 | 238 | WSERIRE | 0.891 |
| 236 | I | 233 | 239 | SERIREF | 0.84 |
| 237 | R | 234 | 240 | ERIREFM | 0.721 |
| 238 | E | 235 | 241 | RIREFME | 0.721 |
| 239 | F | 236 | 242 | IREFMEK | 0.73 |
| 240 | M | 237 | 243 | REFMEKE | 0.769 |
| 241 | E | 238 | 244 | EFMEKEC | 0.803 |
| 242 | K | 239 | 245 | FMEKECP | 0.914 |
| 243 | E | 240 | 246 | MEKECPF | 0.914 |
| 244 | C | 241 | 247 | EKECPFI | 0.896 |
| 245 | P | 242 | 248 | KECPFIK | 0.934 |
| 246 | F | 243 | 249 | ECPFIKP | 1.007 |
| 247 | I | 244 | 250 | CPFIKPE | 1.007 |
| 248 | K | 245 | 251 | PFIKPEV | 0.909 |
| 249 | P | 246 | 252 | FIKPEVK | 0.836 |
| 250 | E | 247 | 253 | IKPEVKP | 0.967 |
| 251 | V | 248 | 254 | KPEVKPG | 1.123 |
| 252 | K | 249 | 255 | PEVKPGT | 1.116 |
| 253 | P | 250 | 256 | EVKPGTP | 1.116 |
| 254 | G | 251 | 257 | VKPGTPA | 1.104 |
| 255 | T | 252 | 258 | KPGTPAQ | 1.173 |
| 256 | P | 253 | 259 | PGTPAQE | 1.134 |
| 257 | A | 254 | 260 | GTPAQEI | 0.984 |
| 258 | Q | 255 | 261 | TPAQEIE | 0.867 |
| 259 | E | 256 | 262 | PAQEIEM | 0.816 |
| 260 | I | 257 | 263 | AQEIEML | 0.683 |
| 261 | E | 258 | 264 | QEIEMLK | 0.733 |
| 262 | M | 259 | 265 | EIEMLKR | 0.729 |
| 263 | L | 260 | 266 | IEMLKRN | 0.846 |
| 264 | K | 261 | 267 | EMLKRNK | 0.923 |
| 265 | R | 262 | 268 | MLKRNKI | 0.884 |
| 266 | N | 263 | 269 | LKRNKIY | 0.961 |
| 267 | K | 264 | 270 | KRNKIYF | 0.963 |
| 268 | I | 265 | 271 | RNKIYFM | 0.904 |
| 269 | Y | 266 | 272 | NKIYFMQ | 0.909 |
| 270 | F | 267 | 273 | KIYFMQR | 0.821 |
| 271 | M | 268 | 274 | IYFMQRQ | 0.817 |
| 272 | Q | 269 | 275 | YFMQRQD | 0.959 |
| 273 | R | 270 | 276 | FMQRQDV | 0.867 |
| 274 | Q | 271 | 277 | MQRQDVL | 0.866 |
| 275 | D | 272 | 278 | QRQDVLD | 0.989 |
| 276 | V | 273 | 279 | RQDVLDK | 0.993 |
| 277 | L | 274 | 280 | QDVLDKN | 1.08 |
| 278 | D | 275 | 281 | DVLDKNH | 1.076 |
| 279 | K | 276 | 282 | VLDKNHV | 0.939 |
| 280 | N | 277 | 283 | LDKNHVA | 0.961 |
| 281 | H | 278 | 284 | DKNHVAD | 1.086 |
| 282 | V | 279 | 285 | KNHVADI | 0.944 |
| 283 | A | 280 | 286 | NHVADID | 1.009 |
| 284 | D | 281 | 287 | HVADIDK | 0.93 |
| 285 | I | 282 | 288 | VADIDKL | 0.879 |
| 286 | D | 283 | 289 | ADIDKLI | 0.874 |
| 287 | K | 284 | 290 | DIDKLID | 0.989 |
| 288 | L | 285 | 291 | IDKLIDY | 0.943 |
| 289 | I | 286 | 292 | DKLIDYA | 0.97 |
| 290 | D | 287 | 293 | KLIDYAA | 0.856 |
| 291 | Y | 288 | 294 | LIDYAAS | 0.916 |
| 292 | A | 289 | 295 | IDYAASG | 1.054 |
| 293 | A | 290 | 296 | DYAASGD | 1.196 |
| 294 | S | 291 | 297 | YAASGDP | 1.204 |
| 295 | G | 292 | 298 | AASGDPT | 1.179 |
| 296 | D | 293 | 299 | ASGDPTS | 1.289 |
| 297 | P | 294 | 300 | SGDPTSP | 1.411 |
| 298 | T | 295 | 301 | GDPTSPD | 1.416 |
| 299 | S | 296 | 302 | DPTSPDN | 1.416 |
| 300 | P | 297 | 303 | PTSPDNI | 1.274 |
| 301 | D | 298 | 304 | TSPDNID | 1.266 |
| 302 | N | 299 | 305 | SPDNIDS | 1.333 |
| 303 | I | 300 | 306 | PDNIDSP | 1.346 |
| 304 | D | 301 | 307 | DNIDSPN | 1.351 |
| 305 | S | 302 | 308 | NIDSPNA | 1.237 |
| 306 | P | 303 | 309 | IDSPNAP | 1.231 |
| 307 | N | 304 | 310 | DSPNAPW | 1.301 |
| 308 | A | 305 | 311 | SPNAPWV | 1.164 |
| 309 | P | 306 | 312 | PNAPWVF | 1.046 |
| 310 | W | 307 | 313 | NAPWVFA | 0.923 |
| 311 | V | 308 | 314 | APWVFAC | 0.87 |
| 312 | F | 309 | 315 | PWVFACA | 0.87 |
| 313 | A | 310 | 316 | WVFACAP | 0.87 |
| 314 | C | 311 | 317 | VFACAPD | 0.941 |
| 315 | A | 312 | 318 | FACAPDR | 1.006 |
| 316 | P | 313 | 319 | ACAPDRC | 1.09 |
| 317 | D | 314 | 320 | CAPDRCP | 1.213 |
| 318 | R | 315 | 321 | APDRCPP | 1.26 |
| 319 | C | 316 | 322 | PDRCPPT | 1.303 |
| 320 | P | 317 | 323 | DRCPPTC | 1.256 |
| 321 | P | 318 | 324 | RCPPTCI | 1.114 |
| 322 | T | 319 | 325 | CPPTCIY | 1.141 |
| 323 | C | 320 | 326 | PPTCIYV | 1.043 |
| 324 | I | 321 | 327 | PTCIYVA | 0.92 |
| 325 | Y | 322 | 328 | TCIYVAG | 0.926 |
| 326 | V | 323 | 329 | CIYVAGM | 0.874 |
| 327 | A | 324 | 330 | IYVAGMA | 0.799 |
| 328 | G | 325 | 331 | YVAGMAE | 0.837 |
| 329 | M | 326 | 332 | VAGMAEL | 0.759 |
| 330 | A | 327 | 333 | AGMAELG | 0.91 |
| 331 | E | 328 | 334 | GMAELGA | 0.91 |
| 332 | L | 329 | 335 | MAELGAF | 0.773 |
| 333 | G | 330 | 336 | AELGAFF | 0.773 |
| 334 | A | 331 | 337 | ELGAFFS | 0.883 |
| 335 | F | 332 | 338 | LGAFFSI | 0.844 |
| 336 | F | 333 | 339 | GAFFSIL | 0.844 |
| 337 | S | 334 | 340 | AFFSILQ | 0.761 |
| 338 | I | 335 | 341 | FFSILQD | 0.876 |
| 339 | L | 336 | 342 | FSILQDM | 0.876 |
| 340 | Q | 337 | 343 | SILQDMR | 0.926 |
| 341 | D | 338 | 344 | ILQDMRN | 0.944 |
| 342 | M | 339 | 345 | LQDMRNT | 1.014 |
| 343 | R | 340 | 346 | QDMRNTI | 0.997 |
| 344 | N | 341 | 347 | DMRNTIM | 0.943 |
| 345 | T | 342 | 348 | MRNTIMA | 0.829 |
| 346 | I | 343 | 349 | RNTIMAS | 0.947 |
| 347 | M | 344 | 350 | NTIMASK | 0.956 |
| 348 | A | 345 | 351 | TIMASKT | 0.87 |
| 349 | S | 346 | 352 | IMASKTV | 0.804 |
| 350 | K | 347 | 353 | MASKTVG | 0.96 |
| 351 | T | 348 | 354 | ASKTVGT | 1.011 |
| 352 | V | 349 | 355 | SKTVGTA | 1.011 |
| 353 | G | 350 | 356 | KTVGTAE | 0.913 |
| 354 | T | 351 | 357 | TVGTAEE | 0.874 |
| 355 | A | 352 | 358 | VGTAEEK | 0.881 |
| 356 | E | 353 | 359 | GTAEEKL | 0.894 |
| 357 | E | 354 | 360 | TAEEKLK | 0.816 |
| 358 | K | 355 | 361 | AEEKLKK | 0.823 |
| 359 | L | 356 | 362 | EEKLKKK | 0.873 |
| 360 | K | 357 | 363 | EKLKKKS | 0.971 |
| 361 | K | 358 | 364 | KLKKKSS | 1.07 |
| 362 | K | 359 | 365 | LKKKSSF | 1.011 |
| 363 | S | 360 | 366 | KKKSSFY | 1.09 |
| 364 | S | 361 | 367 | KKSSFYQ | 1.086 |
| 365 | F | 362 | 368 | KSSFYQS | 1.146 |
| 366 | Y | 363 | 369 | SSFYQSY | 1.164 |
| 367 | Q | 364 | 370 | SFYQSYL | 1.044 |
| 368 | S | 365 | 371 | FYQSYLR | 0.976 |
| 369 | Y | 366 | 372 | YQSYLRR | 1.026 |
| 370 | L | 367 | 373 | QSYLRRT | 1 |
| 371 | R | 368 | 374 | SYLRRTQ | 1 |
| 372 | R | 369 | 375 | YLRRTQS | 1 |
| 373 | T | 370 | 376 | LRRTQSM | 0.923 |
| 374 | Q | 371 | 377 | RRTQSMG | 1.061 |
| 375 | S | 372 | 378 | RTQSMGI | 0.993 |
| 376 | M | 373 | 379 | TQSMGIQ | 0.997 |
| 377 | G | 374 | 380 | QSMGIQL | 0.944 |
| 378 | I | 375 | 381 | SMGIQLD | 1.013 |
| 379 | Q | 376 | 382 | MGIQLDQ | 0.949 |
| 380 | L | 377 | 383 | GIQLDQR | 0.999 |
| 381 | D | 378 | 384 | IQLDQRI | 0.843 |
| 382 | Q | 379 | 385 | QLDQRII | 0.843 |
| 383 | R | 380 | 386 | LDQRIIL | 0.787 |
| 384 | I | 381 | 387 | DQRIILL | 0.787 |
| 385 | I | 382 | 388 | QRIILLF | 0.664 |
| 386 | L | 383 | 389 | RIILLFM | 0.61 |
| 387 | L | 384 | 390 | IILLFML | 0.559 |
| 388 | F | 385 | 391 | ILLFMLE | 0.597 |
| 389 | M | 386 | 392 | LLFMLEW | 0.667 |
| 390 | L | 387 | 393 | LFMLEWG | 0.806 |
| 391 | E | 388 | 394 | FMLEWGK | 0.866 |
| 392 | W | 389 | 395 | MLEWGKE | 0.886 |
| 393 | G | 390 | 396 | LEWGKEM | 0.886 |
| 394 | K | 391 | 397 | EWGKEMV | 0.873 |
| 395 | E | 392 | 398 | WGKEMVD | 0.976 |
| 396 | M | 393 | 399 | GKEMVDH | 0.974 |
| 397 | V | 394 | 400 | KEMVDHF | 0.837 |
| 398 | D | 395 | 401 | EMVDHFH | 0.829 |
| 399 | H | 396 | 402 | MVDHFHL | 0.807 |
| 400 | F | 397 | 403 | VDHFHLG | 0.944 |
| 401 | H | 398 | 404 | DHFHLGD | 1.081 |
| 402 | L | 399 | 405 | HFHLGDD | 1.081 |
| 403 | G | 400 | 406 | FHLGDDM | 1.031 |
| 404 | D | 401 | 407 | HLGDDMD | 1.154 |
| 405 | D | 402 | 408 | LGDDMDP | 1.236 |
| 406 | M | 403 | 409 | GDDMDPE | 1.257 |
| 407 | D | 404 | 410 | DDMDPEL | 1.119 |
| 408 | P | 405 | 411 | DMDPELR | 1.046 |
| 409 | E | 406 | 412 | MDPELRG | 1.06 |
| 410 | L | 407 | 413 | DPELRGL | 1.059 |
| 411 | R | 408 | 414 | PELRGLA | 0.944 |
| 412 | G | 409 | 415 | ELRGLAQ | 0.867 |
| 413 | L | 410 | 416 | LRGLAQA | 0.856 |
| 414 | A | 411 | 417 | RGLAQAL | 0.856 |
| 415 | Q | 412 | 418 | GLAQALI | 0.787 |
| 416 | A | 413 | 419 | LAQALID | 0.773 |
| 417 | L | 414 | 420 | AQALIDQ | 0.829 |
| 418 | I | 415 | 421 | QALIDQK | 0.879 |
| 419 | D | 416 | 422 | ALIDQKV | 0.81 |
| 420 | Q | 417 | 423 | LIDQKVK | 0.86 |
| 421 | K | 418 | 424 | IDQKVKE | 0.881 |
| 422 | V | 419 | 425 | DQKVKEI | 0.881 |
| 423 | K | 420 | 426 | QKVKEIS | 0.877 |
| 424 | E | 421 | 427 | KVKEISN | 0.96 |
| 425 | I | 422 | 428 | VKEISNQ | 0.956 |
| 426 | S | 423 | 429 | KEISNQE | 0.99 |
| 427 | N | 424 | 430 | EISNQEP | 1.063 |
| 428 | Q | 425 | 431 | ISNQEPL | 1.041 |
| 429 | E | 426 | 432 | SNQEPLK | 1.119 |
| 430 | P | 427 | 433 | NQEPLKI | 0.981 |
| **Seoul orthohantavirus** | | | | | |
| 4 | M | 1 | 7 | MATMEEI | 0.681 |
| 5 | E | 2 | 8 | ATMEEIQ | 0.736 |
| 6 | E | 3 | 9 | TMEEIQR | 0.777 |
| 7 | I | 4 | 10 | MEEIQRE | 0.746 |
| 8 | Q | 5 | 11 | EEIQREI | 0.727 |
| 9 | R | 6 | 12 | EIQREIS | 0.826 |
| 10 | E | 7 | 13 | IQREISA | 0.814 |
| 11 | I | 8 | 14 | QREISAH | 0.883 |
| 12 | S | 9 | 15 | REISAHE | 0.849 |
| 13 | A | 10 | 16 | EISAHEG | 0.936 |
| 14 | H | 11 | 17 | ISAHEGQ | 0.97 |
| 15 | E | 12 | 18 | SAHEGQL | 0.987 |
| 16 | G | 13 | 19 | AHEGQLV | 0.854 |
| 17 | Q | 14 | 20 | HEGQLVI | 0.827 |
| 18 | L | 15 | 21 | EGQLVIA | 0.786 |
| 19 | V | 16 | 22 | GQLVIAR | 0.816 |
| 20 | I | 17 | 23 | QLVIARQ | 0.733 |
| 21 | A | 18 | 24 | LVIARQN | 0.816 |
| 22 | R | 19 | 25 | VIARQNV | 0.803 |
| 23 | Q | 20 | 26 | IARQNVK | 0.876 |
| 24 | N | 21 | 27 | ARQNVKD | 1.017 |
| 25 | V | 22 | 28 | RQNVKDA | 1.017 |
| 26 | K | 23 | 29 | QNVKDAE | 0.987 |
| 27 | D | 24 | 30 | NVKDAEK | 0.991 |
| 28 | A | 25 | 31 | VKDAEKQ | 0.909 |
| 29 | E | 26 | 32 | KDAEKQY | 1 |
| 30 | K | 27 | 33 | DAEKQYE | 0.961 |
| 31 | Q | 28 | 34 | AEKQYEK | 0.897 |
| 32 | Y | 29 | 35 | EKQYEKD | 1.011 |
| 33 | E | 30 | 36 | KQYEKDP | 1.123 |
| 34 | K | 31 | 37 | QYEKDPD | 1.187 |
| 35 | D | 32 | 38 | YEKDPDD | 1.256 |
| 36 | P | 33 | 39 | EKDPDDL | 1.177 |
| 37 | D | 34 | 40 | KDPDDLN | 1.294 |
| 38 | D | 35 | 41 | DPDDLNK | 1.294 |
| 39 | L | 36 | 42 | PDDLNKR | 1.221 |
| 40 | N | 37 | 43 | DDLNKRA | 1.099 |
| 41 | K | 38 | 44 | DLNKRAL | 0.974 |
| 42 | R | 39 | 45 | LNKRALH | 0.901 |
| 43 | A | 40 | 46 | NKRALHD | 1.026 |
| 44 | L | 41 | 47 | KRALHDR | 0.939 |
| 45 | H | 42 | 48 | RALHDRE | 0.9 |
| 46 | D | 43 | 49 | ALHDRES | 0.969 |
| 47 | R | 44 | 50 | LHDRESV | 0.946 |
| 48 | E | 45 | 51 | HDRESVA | 0.956 |
| 49 | S | 46 | 52 | DRESVAA | 0.914 |
| 50 | V | 47 | 53 | RESVAAS | 0.91 |
| 51 | A | 48 | 54 | ESVAASI | 0.841 |
| 52 | A | 49 | 55 | SVAASIQ | 0.876 |
| 53 | S | 50 | 56 | VAASIQS | 0.876 |
| 54 | I | 51 | 57 | AASIQSK | 0.949 |
| 55 | Q | 52 | 58 | ASIQSKI | 0.921 |
| 56 | S | 53 | 59 | SIQSKID | 1.036 |
| 57 | K | 54 | 60 | IQSKIDE | 0.937 |
| 58 | I | 55 | 61 | QSKIDEL | 0.954 |
| 59 | D | 56 | 62 | SKIDELK | 0.959 |
| 60 | E | 57 | 63 | KIDELKR | 0.89 |
| 61 | L | 58 | 64 | IDELKRQ | 0.886 |
| 62 | K | 59 | 65 | DELKRQP | 1.036 |
| 63 | R | 60 | 66 | ELKRQPA | 0.921 |
| 64 | Q | 61 | 67 | LKRQPAD | 1.024 |
| 65 | P | 62 | 68 | KRQPADR | 1.076 |
| 66 | A | 63 | 69 | RQPADRI | 0.999 |
| 67 | D | 64 | 70 | QPADRIA | 0.957 |
| 68 | R | 65 | 71 | PADRIAA | 0.911 |
| 69 | I | 66 | 72 | ADRIAAG | 0.917 |
| 70 | A | 67 | 73 | DRIAAGK | 0.967 |
| 71 | A | 68 | 74 | RIAAGKN | 0.981 |
| 72 | G | 69 | 75 | IAAGKNI | 0.913 |
| 73 | K | 70 | 76 | AAGKNIG | 1.069 |
| 74 | N | 71 | 77 | AGKNIGQ | 1.114 |
| 75 | I | 72 | 78 | GKNIGQD | 1.229 |
| 76 | G | 73 | 79 | KNIGQDR | 1.141 |
| 77 | Q | 74 | 80 | NIGQDRD | 1.206 |
| 78 | D | 75 | 81 | IGQDRDP | 1.2 |
| 79 | R | 76 | 82 | GQDRDPT | 1.27 |
| 80 | D | 77 | 83 | QDRDPTG | 1.27 |
| 81 | P | 78 | 84 | DRDPTGV | 1.201 |
| 82 | T | 79 | 85 | RDPTGVE | 1.099 |
| 83 | G | 80 | 86 | DPTGVEP | 1.18 |
| 84 | V | 81 | 87 | PTGVEPG | 1.194 |
| 85 | E | 82 | 88 | TGVEPGD | 1.186 |
| 86 | P | 83 | 89 | GVEPGDH | 1.184 |
| 87 | G | 84 | 90 | VEPGDHL | 1.046 |
| 88 | D | 85 | 91 | EPGDHLK | 1.119 |
| 89 | H | 86 | 92 | PGDHLKE | 1.119 |
| 90 | L | 87 | 93 | GDHLKER | 1.037 |
| 91 | K | 88 | 94 | DHLKERS | 1.019 |
| 92 | E | 89 | 95 | HLKERSA | 0.904 |
| 93 | R | 90 | 96 | LKERSAL | 0.853 |
| 94 | S | 91 | 97 | KERSALS | 0.973 |
| 95 | A | 92 | 98 | ERSALSY | 0.991 |
| 96 | L | 93 | 99 | RSALSYG | 1.109 |
| 97 | S | 94 | 100 | SALSYGN | 1.196 |
| 98 | Y | 95 | 101 | ALSYGNT | 1.129 |
| 99 | G | 96 | 102 | LSYGNTL | 1.119 |
| 100 | N | 97 | 103 | SYGNTLD | 1.243 |
| 101 | T | 98 | 104 | YGNTLDL | 1.123 |
| 102 | L | 99 | 105 | GNTLDLN | 1.183 |
| 103 | D | 100 | 106 | NTLDLNR | 1.096 |
| 104 | L | 101 | 107 | TLDLNRL | 0.957 |
| 105 | N | 102 | 108 | LDLNRLD | 1.029 |
| 106 | R | 103 | 109 | DLNRLDI | 1.011 |
| 107 | L | 104 | 110 | LNRLDID | 1.011 |
| 108 | D | 105 | 111 | NRLDIDE | 1.033 |
| 109 | I | 106 | 112 | RLDIDEP | 1.027 |
| 110 | D | 107 | 113 | LDIDEPT | 1.029 |
| 111 | E | 108 | 114 | DIDEPTG | 1.167 |
| 112 | P | 109 | 115 | IDEPTGQ | 1.099 |
| 113 | T | 110 | 116 | DEPTGQR | 1.167 |
| 114 | G | 111 | 117 | EPTGQRA | 1.053 |
| 115 | Q | 112 | 118 | PTGQRAD | 1.156 |
| 116 | R | 113 | 119 | TGQRADW | 1.076 |
| 117 | A | 114 | 120 | GQRADWL | 1.023 |
| 118 | D | 115 | 121 | QRADWLT | 0.937 |
| 119 | W | 116 | 122 | RADWLTI | 0.864 |
| 120 | L | 117 | 123 | ADWLTII | 0.796 |
| 121 | T | 118 | 124 | DWLTIIV | 0.773 |
| 122 | I | 119 | 125 | WLTIIVY | 0.727 |
| 123 | I | 120 | 126 | LTIIVYL | 0.674 |
| 124 | V | 121 | 127 | TIIVYLT | 0.727 |
| 125 | Y | 122 | 128 | IIVYLTS | 0.794 |
| 126 | L | 123 | 129 | IVYLTSF | 0.813 |
| 127 | T | 124 | 130 | VYLTSFV | 0.817 |
| 128 | S | 125 | 131 | YLTSFVV | 0.817 |
| 129 | F | 126 | 132 | LTSFVVR | 0.79 |
| 130 | V | 127 | 133 | TSFVVRI | 0.773 |
| 131 | V | 128 | 134 | SFVVRII | 0.703 |
| 132 | R | 129 | 135 | FVVRIIL | 0.583 |
| 133 | I | 130 | 136 | VVRIILK | 0.641 |
| 134 | I | 131 | 137 | VRIILKA | 0.664 |
| 135 | L | 132 | 138 | RIILKAL | 0.677 |
| 136 | K | 133 | 139 | IILKALY | 0.704 |
| 137 | A | 134 | 140 | ILKALYM | 0.723 |
| 138 | L | 135 | 141 | LKALYMF | 0.741 |
| 139 | Y | 136 | 142 | KALYMFS | 0.861 |
| 140 | M | 137 | 143 | ALYMFST | 0.854 |
| 141 | F | 138 | 144 | LYMFSTR | 0.896 |
| 142 | S | 139 | 145 | YMFSTRG | 1.034 |
| 143 | T | 140 | 146 | MFSTRGR | 1.007 |
| 144 | R | 141 | 147 | FSTRGRQ | 1.061 |
| 145 | G | 142 | 148 | STRGRQT | 1.113 |
| 146 | R | 143 | 149 | TRGRQTS | 1.113 |
| 147 | Q | 144 | 150 | RGRQTSK | 1.12 |
| 148 | T | 145 | 151 | GRQTSKD | 1.193 |
| 149 | S | 146 | 152 | RQTSKDN | 1.193 |
| 150 | K | 147 | 153 | QTSKDNK | 1.201 |
| 151 | D | 148 | 154 | TSKDNKG | 1.284 |
| 152 | N | 149 | 155 | SKDNKGL | 1.231 |
| 153 | K | 150 | 156 | KDNKGLR | 1.163 |
| 154 | G | 151 | 157 | DNKGLRI | 1.086 |
| 155 | L | 152 | 158 | NKGLRIR | 1.013 |
| 156 | R | 153 | 159 | KGLRIRF | 0.876 |
| 157 | I | 154 | 160 | GLRIRFK | 0.876 |
| 158 | R | 155 | 161 | LRIRFKD | 0.861 |
| 159 | F | 156 | 162 | RIRFKDD | 0.986 |
| 160 | K | 157 | 163 | IRFKDDS | 1.054 |
| 161 | D | 158 | 164 | RFKDDSS | 1.191 |
| 162 | D | 159 | 165 | FKDDSSY | 1.219 |
| 163 | S | 160 | 166 | KDDSSYE | 1.239 |
| 164 | S | 161 | 167 | DDSSYED | 1.303 |
| 165 | Y | 162 | 168 | DSSYEDF | 1.18 |
| 166 | E | 163 | 169 | SSYEDFN | 1.194 |
| 167 | D | 164 | 170 | SYEDFNG | 1.213 |
| 168 | F | 165 | 171 | YEDFNGI | 1.076 |
| 169 | N | 166 | 172 | EDFNGIR | 1.049 |
| 170 | G | 167 | 173 | DFNGIRK | 1.087 |
| 171 | I | 168 | 174 | FNGIRKP | 1.096 |
| 172 | R | 169 | 175 | NGIRKPK | 1.154 |
| 173 | K | 170 | 176 | GIRKPKH | 1.067 |
| 174 | P | 171 | 177 | IRKPKHL | 0.929 |
| 175 | K | 172 | 178 | RKPKHLY | 1.024 |
| 176 | H | 173 | 179 | KPKHLYV | 0.96 |
| 177 | L | 174 | 180 | PKHLYVP | 1.033 |
| 178 | Y | 175 | 181 | KHLYVPM | 0.901 |
| 179 | V | 176 | 182 | HLYVPMP | 0.974 |
| 180 | P | 177 | 183 | LYVPMPN | 1.061 |
| 181 | M | 178 | 184 | YVPMPNA | 1.071 |
| 182 | P | 179 | 185 | VPMPNAQ | 1.049 |
| 183 | N | 180 | 186 | PMPNAQS | 1.181 |
| 184 | A | 181 | 187 | MPNAQSS | 1.169 |
| 185 | Q | 182 | 188 | PNAQSSM | 1.169 |
| 186 | S | 183 | 189 | NAQSSMK | 1.096 |
| 187 | S | 184 | 190 | AQSSMKA | 0.967 |
| 188 | M | 185 | 191 | QSSMKAE | 0.979 |
| 189 | K | 186 | 192 | SSMKAEE | 0.944 |
| 190 | A | 187 | 193 | SMKAEEI | 0.807 |
| 191 | E | 188 | 194 | MKAEEIT | 0.74 |
| 192 | E | 189 | 195 | KAEEITP | 0.871 |
| 193 | I | 190 | 196 | AEEITPG | 0.95 |
| 194 | T | 191 | 197 | EEITPGR | 0.991 |
| 195 | P | 192 | 198 | EITPGRF | 0.971 |
| 196 | G | 193 | 199 | ITPGRFR | 1.001 |
| 197 | R | 194 | 200 | TPGRFRT | 1.071 |
| 198 | F | 195 | 201 | PGRFRTA | 1.029 |
| 199 | R | 196 | 202 | GRFRTAV | 0.883 |
| 200 | T | 197 | 203 | RFRTAVC | 0.83 |
| 201 | A | 198 | 204 | FRTAVCG | 0.917 |
| 202 | V | 199 | 205 | RTAVCGL | 0.916 |
| 203 | C | 200 | 206 | TAVCGLY | 0.943 |
| 204 | G | 201 | 207 | AVCGLYP | 1.023 |
| 205 | L | 202 | 208 | VCGLYPA | 1.023 |
| 206 | Y | 203 | 209 | CGLYPAQ | 1.091 |
| 207 | P | 204 | 210 | GLYPAQI | 0.989 |
| 208 | A | 205 | 211 | LYPAQIK | 0.91 |
| 209 | Q | 206 | 212 | YPAQIKA | 0.92 |
| 210 | I | 207 | 213 | PAQIKAR | 0.893 |
| 211 | K | 208 | 214 | AQIKARN | 0.899 |
| 212 | A | 209 | 215 | QIKARNM | 0.89 |
| 213 | R | 210 | 216 | IKARNMV | 0.821 |
| 214 | N | 211 | 217 | KARNMVS | 0.959 |
| 215 | M | 212 | 218 | ARNMVSP | 1.031 |
| 216 | V | 213 | 219 | RNMVSPV | 1.009 |
| 217 | S | 214 | 220 | NMVSPVM | 0.959 |
| 218 | P | 215 | 221 | MVSPVMS | 0.94 |
| 219 | V | 216 | 222 | VSPVMSV | 0.926 |
| 220 | M | 217 | 223 | SPVMSVV | 0.926 |
| 221 | S | 218 | 224 | PVMSVVG | 0.944 |
| 222 | V | 219 | 225 | VMSVVGF | 0.813 |
| 223 | V | 220 | 226 | MSVVGFL | 0.826 |
| 224 | G | 221 | 227 | SVVGFLA | 0.834 |
| 225 | F | 222 | 228 | VVGFLAL | 0.714 |
| 226 | L | 223 | 229 | VGFLALA | 0.737 |
| 227 | A | 224 | 230 | GFLALAK | 0.81 |
| 228 | L | 225 | 231 | FLALAKD | 0.796 |
| 229 | A | 226 | 232 | LALAKDW | 0.847 |
| 230 | K | 227 | 233 | ALAKDWT | 0.9 |
| 231 | D | 228 | 234 | LAKDWTS | 1.01 |
| 232 | W | 229 | 235 | AKDWTSR | 1.061 |
| 233 | T | 230 | 236 | KDWTSRI | 1.034 |
| 234 | S | 231 | 237 | DWTSRIE | 0.996 |
| 235 | R | 232 | 238 | WTSRIEE | 0.893 |
| 236 | I | 233 | 239 | TSRIEEW | 0.893 |
| 237 | E | 234 | 240 | SRIEEWL | 0.84 |
| 238 | E | 235 | 241 | RIEEWLG | 0.859 |
| 239 | W | 236 | 242 | IEEWLGA | 0.817 |
| 240 | L | 237 | 243 | EEWLGAP | 0.967 |
| 241 | G | 238 | 244 | EWLGAPC | 1.031 |
| 242 | A | 239 | 245 | WLGAPCK | 1.07 |
| 243 | P | 240 | 246 | LGAPCKF | 1.019 |
| 244 | C | 241 | 247 | GAPCKFM | 1.02 |
| 245 | K | 242 | 248 | APCKFMA | 0.891 |
| 246 | F | 243 | 249 | PCKFMAE | 0.903 |
| 247 | M | 244 | 250 | CKFMAES | 0.89 |
| 248 | A | 245 | 251 | KFMAESP | 0.937 |
| 249 | E | 246 | 252 | FMAESPI | 0.86 |
| 250 | S | 247 | 253 | MAESPIA | 0.869 |
| 251 | P | 248 | 254 | AESPIAG | 1.006 |
| 252 | I | 249 | 255 | ESPIAGS | 1.116 |
| 253 | A | 250 | 256 | SPIAGSL | 1.094 |
| 254 | G | 251 | 257 | PIAGSLS | 1.094 |
| 255 | S | 252 | 258 | IAGSLSG | 1.1 |
| 256 | L | 253 | 259 | AGSLSGN | 1.256 |
| 257 | S | 254 | 260 | GSLSGNP | 1.379 |
| 258 | G | 255 | 261 | SLSGNPV | 1.227 |
| 259 | N | 256 | 262 | LSGNPVN | 1.246 |
| 260 | P | 257 | 263 | SGNPVNR | 1.297 |
| 261 | V | 258 | 264 | GNPVNRD | 1.301 |
| 262 | N | 259 | 265 | NPVNRDY | 1.241 |
| 263 | R | 260 | 266 | PVNRDYI | 1.086 |
| 264 | D | 261 | 267 | VNRDYIR | 1.004 |
| 265 | Y | 262 | 268 | NRDYIRQ | 1.073 |
| 266 | I | 263 | 269 | RDYIRQR | 0.986 |
| 267 | R | 264 | 270 | DYIRQRQ | 0.99 |
| 268 | Q | 265 | 271 | YIRQRQG | 1.004 |
| 269 | R | 266 | 272 | IRQRQGA | 0.936 |
| 270 | Q | 267 | 273 | RQRQGAL | 0.953 |
| 271 | G | 268 | 274 | QRQGALA | 0.911 |
| 272 | A | 269 | 275 | RQGALAA | 0.866 |
| 273 | L | 270 | 276 | QGALAAM | 0.816 |
| 274 | A | 271 | 277 | GALAAME | 0.781 |
| 275 | A | 272 | 278 | ALAAMEP | 0.776 |
| 276 | M | 273 | 279 | LAAMEPK | 0.826 |
| 277 | E | 274 | 280 | AAMEPKE | 0.847 |
| 278 | P | 275 | 281 | AMEPKEF | 0.839 |
| 279 | K | 276 | 282 | MEPKEFQ | 0.884 |
| 280 | E | 277 | 283 | EPKEFQA | 0.893 |
| 281 | F | 278 | 284 | PKEFQAL | 0.871 |
| 282 | Q | 279 | 285 | KEFQALR | 0.79 |
| 283 | A | 280 | 286 | EFQALRQ | 0.786 |
| 284 | L | 281 | 287 | FQALRQH | 0.816 |
| 285 | R | 282 | 288 | QALRQHA | 0.824 |
| 286 | Q | 283 | 289 | ALRQHAK | 0.829 |
| 287 | H | 284 | 290 | LRQHAKD | 0.943 |
| 288 | A | 285 | 291 | RQHAKDP | 1.076 |
| 289 | K | 286 | 292 | QHAKDPG | 1.163 |
| 290 | D | 287 | 293 | HAKDPGC | 1.193 |
| 291 | P | 288 | 294 | AKDPGCT | 1.194 |
| 292 | G | 289 | 295 | KDPGCTL | 1.184 |
| 293 | C | 290 | 296 | DPGCTLV | 1.111 |
| 294 | T | 291 | 297 | PGCTLVE | 1.009 |
| 295 | L | 292 | 298 | GCTLVEH | 0.927 |
| 296 | V | 293 | 299 | CTLVEHI | 0.771 |
| 297 | E | 294 | 300 | TLVEHIE | 0.707 |
| 298 | H | 295 | 301 | LVEHIES | 0.774 |
| 299 | I | 296 | 302 | VEHIESP | 0.907 |
| 300 | E | 297 | 303 | EHIESPS | 1.04 |
| 301 | S | 298 | 304 | HIESPSS | 1.139 |
| 302 | P | 299 | 305 | IESPSSI | 1.07 |
| 303 | S | 300 | 306 | ESPSSIW | 1.14 |
| 304 | S | 301 | 307 | SPSSIWV | 1.106 |
| 305 | I | 302 | 308 | PSSIWVS | 1.106 |
| 306 | W | 303 | 309 | SSIWVSA | 0.983 |
| 307 | V | 304 | 310 | SIWVSAG | 1.001 |
| 308 | S | 305 | 311 | IWVSAGA | 0.891 |
| 309 | A | 306 | 312 | WVSAGAP | 1.041 |
| 310 | G | 307 | 313 | VSAGAPD | 1.113 |
| 311 | A | 308 | 314 | SAGAPDR | 1.177 |
| 312 | P | 309 | 315 | AGAPDRC | 1.143 |
| 313 | D | 310 | 316 | GAPDRCP | 1.266 |
| 314 | R | 311 | 317 | APDRCPP | 1.26 |
| 315 | C | 312 | 318 | PDRCPPT | 1.303 |
| 316 | P | 313 | 319 | DRCPPTC | 1.256 |
| 317 | P | 314 | 320 | RCPPTCL | 1.131 |
| 318 | T | 315 | 321 | CPPTCLF | 1.081 |
| 319 | C | 316 | 322 | PPTCLFV | 0.983 |
| 320 | L | 317 | 323 | PTCLFVG | 0.989 |
| 321 | F | 318 | 324 | TCLFVGG | 0.994 |
| 322 | V | 319 | 325 | CLFVGGM | 0.943 |
| 323 | G | 320 | 326 | LFVGGMA | 0.867 |
| 324 | G | 321 | 327 | FVGGMAE | 0.889 |
| 325 | M | 322 | 328 | VGGMAEL | 0.887 |
| 326 | A | 323 | 329 | GGMAELG | 1.039 |
| 327 | E | 324 | 330 | GMAELGA | 0.91 |
| 328 | L | 325 | 331 | MAELGAF | 0.773 |
| 329 | G | 326 | 332 | AELGAFF | 0.773 |
| 330 | A | 327 | 333 | ELGAFFS | 0.883 |
| 331 | F | 328 | 334 | LGAFFSI | 0.844 |
| 332 | F | 329 | 335 | GAFFSIF | 0.846 |
| 333 | S | 330 | 336 | AFFSIFQ | 0.763 |
| 334 | I | 331 | 337 | FFSIFQD | 0.877 |
| 335 | F | 332 | 338 | FSIFQDM | 0.877 |
| 336 | Q | 333 | 339 | SIFQDMR | 0.927 |
| 337 | D | 334 | 340 | IFQDMRN | 0.946 |
| 338 | M | 335 | 341 | FQDMRNT | 1.016 |
| 339 | R | 336 | 342 | QDMRNTI | 0.997 |
| 340 | N | 337 | 343 | DMRNTIM | 0.943 |
| 341 | T | 338 | 344 | MRNTIMP | 0.951 |
| 342 | I | 339 | 345 | RNTIMPS | 1.07 |
| 343 | M | 340 | 346 | NTIMPSK | 1.079 |
| 344 | P | 341 | 347 | TIMPSKT | 0.993 |
| 345 | S | 342 | 348 | IMPSKTV | 0.927 |
| 346 | K | 343 | 349 | MPSKTVG | 1.083 |
| 347 | T | 344 | 350 | PSKTVGT | 1.134 |
| 348 | V | 345 | 351 | SKTVGTA | 1.011 |
| 349 | G | 346 | 352 | KTVGTAD | 1.016 |
| 350 | T | 347 | 353 | TVGTADE | 0.977 |
| 351 | A | 348 | 354 | VGTADEK | 0.984 |
| 352 | D | 349 | 355 | GTADEKL | 0.997 |
| 353 | E | 350 | 356 | TADEKLR | 0.91 |
| 354 | K | 351 | 357 | ADEKLRK | 0.917 |
| 355 | L | 352 | 358 | DEKLRKK | 0.967 |
| 356 | R | 353 | 359 | EKLRKKS | 0.963 |
| 357 | K | 354 | 360 | KLRKKSL | 0.941 |
| 358 | K | 355 | 361 | LRKKSLF | 0.883 |
| 359 | S | 356 | 362 | RKKSLFY | 0.961 |
| 360 | L | 357 | 363 | KKSLFYQ | 0.966 |
| 361 | F | 358 | 364 | KSLFYQS | 1.026 |
| 362 | Y | 359 | 365 | SLFYQSY | 1.044 |
| 363 | Q | 360 | 366 | LFYQSYL | 0.924 |
| 364 | S | 361 | 367 | FYQSYLR | 0.976 |
| 365 | Y | 362 | 368 | YQSYLRR | 1.026 |
| 366 | L | 363 | 369 | QSYLRRT | 1 |
| 367 | R | 364 | 370 | SYLRRTQ | 1 |
| 368 | R | 365 | 371 | YLRRTQS | 1 |
| 369 | T | 366 | 372 | LRRTQSM | 0.923 |
| 370 | Q | 367 | 373 | RRTQSMG | 1.061 |
| 371 | S | 368 | 374 | RTQSMGI | 0.993 |
| 372 | M | 369 | 375 | TQSMGIQ | 0.997 |
| 373 | G | 370 | 376 | QSMGIQL | 0.944 |
| 374 | I | 371 | 377 | SMGIQLD | 1.013 |
| 375 | Q | 372 | 378 | MGIQLDQ | 0.949 |
| 376 | L | 373 | 379 | GIQLDQR | 0.999 |
| 377 | D | 374 | 380 | IQLDQRI | 0.843 |
| 378 | Q | 375 | 381 | QLDQRII | 0.843 |
| 379 | R | 376 | 382 | LDQRIIV | 0.774 |
| 380 | I | 377 | 383 | DQRIIVM | 0.776 |
| 381 | I | 378 | 384 | QRIIVMF | 0.653 |
| 382 | V | 379 | 385 | RIIVMFM | 0.599 |
| 383 | M | 380 | 386 | IIVMFMV | 0.534 |
| 384 | F | 381 | 387 | IVMFMVA | 0.561 |
| 385 | M | 382 | 388 | VMFMVAW | 0.631 |
| 386 | V | 383 | 389 | MFMVAWG | 0.783 |
| 387 | A | 384 | 390 | FMVAWGK | 0.841 |
| 388 | W | 385 | 391 | MVAWGKE | 0.861 |
| 389 | G | 386 | 392 | VAWGKEA | 0.87 |
| 390 | K | 387 | 393 | AWGKEAV | 0.87 |
| 391 | E | 388 | 394 | WGKEAVD | 0.984 |
| 392 | A | 389 | 395 | GKEAVDN | 1.07 |
| 393 | V | 390 | 396 | KEAVDNF | 0.933 |
| 394 | D | 391 | 397 | EAVDNFH | 0.924 |
| 395 | N | 392 | 398 | AVDNFHL | 0.903 |
| 396 | F | 393 | 399 | VDNFHLP | 1.026 |
| 397 | H | 394 | 400 | DNFHLPD | 1.163 |
| 398 | L | 395 | 401 | NFHLPDD | 1.163 |
| 399 | P | 396 | 402 | FHLPDDM | 1.026 |
| 400 | D | 397 | 403 | HLPDDMD | 1.149 |
| 401 | D | 398 | 404 | LPDDMDP | 1.23 |
| 402 | M | 399 | 405 | PDDMDPE | 1.251 |
| 403 | D | 400 | 406 | DDMDPEL | 1.119 |
| 404 | P | 401 | 407 | DMDPELR | 1.046 |
| 405 | E | 402 | 408 | MDPELRS | 1.041 |
| 406 | L | 403 | 409 | DPELRSL | 1.04 |
| 407 | R | 404 | 410 | PELRSLA | 0.926 |
| 408 | S | 405 | 411 | ELRSLAQ | 0.849 |
| 409 | L | 406 | 412 | LRSLAQI | 0.81 |
| 410 | A | 407 | 413 | RSLAQIL | 0.81 |
| 411 | Q | 408 | 414 | SLAQILI | 0.741 |
| 412 | I | 409 | 415 | LAQILIV | 0.609 |
| 413 | L | 410 | 416 | AQILIVQ | 0.664 |
| 414 | I | 411 | 417 | QILIVQK | 0.714 |
| 415 | V | 412 | 418 | ILIVQKV | 0.646 |
| 416 | Q | 413 | 419 | LIVQKVK | 0.723 |
| 417 | K | 414 | 420 | IVQKVKE | 0.744 |
| 418 | V | 415 | 421 | VQKVKEI | 0.744 |
| 419 | K | 416 | 422 | QKVKEIS | 0.877 |
| 420 | E | 417 | 423 | KVKEISN | 0.96 |
| 421 | I | 418 | 424 | VKEISNQ | 0.956 |
| 422 | S | 419 | 425 | KEISNQE | 0.99 |
| 423 | N | 420 | 426 | EISNQEP | 1.063 |
| 424 | Q | 421 | 427 | ISNQEPM | 1.043 |
| 425 | E | 422 | 428 | SNQEPMK | 1.12 |
| 426 | P | 423 | 429 | NQEPMKL | 1 |
| **Dobrava-Belgrade orthohantavirus** | | | | | |
| 4 | L | 1 | 7 | MATLEEL | 0.697 |
| 5 | E | 2 | 8 | ATLEELQ | 0.751 |
| 6 | E | 3 | 9 | TLEELQK | 0.801 |
| 7 | L | 4 | 10 | LEELQKE | 0.77 |
| 8 | Q | 5 | 11 | EELQKEI | 0.753 |
| 9 | K | 6 | 12 | ELQKEIN | 0.87 |
| 10 | E | 7 | 13 | LQKEINN | 0.987 |
| 11 | I | 8 | 14 | QKEINNH | 1.039 |
| 12 | N | 9 | 15 | KEINNHE | 1.004 |
| 13 | N | 10 | 16 | EINNHEG | 1.083 |
| 14 | H | 11 | 17 | INNHEGQ | 1.117 |
| 15 | E | 12 | 18 | NNHEGQL | 1.134 |
| 16 | G | 13 | 19 | NHEGQLV | 0.983 |
| 17 | Q | 14 | 20 | HEGQLVI | 0.827 |
| 18 | L | 15 | 21 | EGQLVIA | 0.786 |
| 19 | V | 16 | 22 | GQLVIAR | 0.816 |
| 20 | I | 17 | 23 | QLVIARQ | 0.733 |
| 21 | A | 18 | 24 | LVIARQK | 0.737 |
| 22 | R | 19 | 25 | VIARQKV | 0.724 |
| 23 | Q | 20 | 26 | IARQKVK | 0.797 |
| 24 | K | 21 | 27 | ARQKVKD | 0.939 |
| 25 | V | 22 | 28 | RQKVKDA | 0.939 |
| 26 | K | 23 | 29 | QKVKDAE | 0.909 |
| 27 | D | 24 | 30 | KVKDAEK | 0.913 |
| 28 | A | 25 | 31 | VKDAEKQ | 0.909 |
| 29 | E | 26 | 32 | KDAEKQY | 1 |
| 30 | K | 27 | 33 | DAEKQYE | 0.961 |
| 31 | Q | 28 | 34 | AEKQYEK | 0.897 |
| 32 | Y | 29 | 35 | EKQYEKD | 1.011 |
| 33 | E | 30 | 36 | KQYEKDP | 1.123 |
| 34 | K | 31 | 37 | QYEKDPD | 1.187 |
| 35 | D | 32 | 38 | YEKDPDD | 1.256 |
| 36 | P | 33 | 39 | EKDPDDL | 1.177 |
| 37 | D | 34 | 40 | KDPDDLN | 1.294 |
| 38 | D | 35 | 41 | DPDDLNK | 1.294 |
| 39 | L | 36 | 42 | PDDLNKR | 1.221 |
| 40 | N | 37 | 43 | DDLNKRA | 1.099 |
| 41 | K | 38 | 44 | DLNKRAL | 0.974 |
| 42 | R | 39 | 45 | LNKRALS | 0.97 |
| 43 | A | 40 | 46 | NKRALSD | 1.094 |
| 44 | L | 41 | 47 | KRALSDR | 1.007 |
| 45 | S | 42 | 48 | RALSDRE | 0.969 |
| 46 | D | 43 | 49 | ALSDRES | 1.037 |
| 47 | R | 44 | 50 | LSDRESI | 1.01 |
| 48 | E | 45 | 51 | SDRESIA | 1.02 |
| 49 | S | 46 | 52 | DRESIAQ | 0.956 |
| 50 | I | 47 | 53 | RESIAQS | 0.951 |
| 51 | A | 48 | 54 | ESIAQSI | 0.883 |
| 52 | Q | 49 | 55 | SIAQSIQ | 0.917 |
| 53 | S | 50 | 56 | IAQSIQG | 0.936 |
| 54 | I | 51 | 57 | AQSIQGK | 1.013 |
| 55 | Q | 52 | 58 | QSIQGKI | 0.986 |
| 56 | G | 53 | 59 | SIQGKID | 1.054 |
| 57 | K | 54 | 60 | IQGKIDE | 0.956 |
| 58 | I | 55 | 61 | QGKIDEL | 0.973 |
| 59 | D | 56 | 62 | GKIDELR | 0.969 |
| 60 | E | 57 | 63 | KIDELRR | 0.881 |
| 61 | L | 58 | 64 | IDELRRQ | 0.877 |
| 62 | R | 59 | 65 | DELRRQL | 0.894 |
| 63 | R | 60 | 66 | ELRRQLA | 0.78 |
| 64 | Q | 61 | 67 | LRRQLAD | 0.883 |
| 65 | L | 62 | 68 | RRQLADR | 0.934 |
| 66 | A | 63 | 69 | RQLADRV | 0.87 |
| 67 | D | 64 | 70 | QLADRVA | 0.829 |
| 68 | R | 65 | 71 | LADRVAA | 0.783 |
| 69 | V | 66 | 72 | ADRVAAG | 0.921 |
| 70 | A | 67 | 73 | DRVAAGK | 0.971 |
| 71 | A | 68 | 74 | RVAAGKN | 0.986 |
| 72 | G | 69 | 75 | VAAGKNI | 0.917 |
| 73 | K | 70 | 76 | AAGKNIG | 1.069 |
| 74 | N | 71 | 77 | AGKNIGK | 1.119 |
| 75 | I | 72 | 78 | GKNIGKE | 1.13 |
| 76 | G | 73 | 79 | KNIGKER | 1.043 |
| 77 | K | 74 | 80 | NIGKERD | 1.107 |
| 78 | E | 75 | 81 | IGKERDP | 1.101 |
| 79 | R | 76 | 82 | GKERDPT | 1.171 |
| 80 | D | 77 | 83 | KERDPTG | 1.171 |
| 81 | P | 78 | 84 | ERDPTGL | 1.111 |
| 82 | T | 79 | 85 | RDPTGLD | 1.214 |
| 83 | G | 80 | 86 | DPTGLDP | 1.296 |
| 84 | L | 81 | 87 | PTGLDPG | 1.31 |
| 85 | D | 82 | 88 | TGLDPGD | 1.301 |
| 86 | P | 83 | 89 | GLDPGDH | 1.3 |
| 87 | G | 84 | 90 | LDPGDHL | 1.161 |
| 88 | D | 85 | 91 | DPGDHLK | 1.221 |
| 89 | H | 86 | 92 | PGDHLKE | 1.119 |
| 90 | L | 87 | 93 | GDHLKEK | 1.046 |
| 91 | K | 88 | 94 | DHLKEKS | 1.027 |
| 92 | E | 89 | 95 | HLKEKSM | 0.904 |
| 93 | K | 90 | 96 | LKEKSML | 0.853 |
| 94 | S | 91 | 97 | KEKSMLS | 0.973 |
| 95 | M | 92 | 98 | EKSMLSY | 0.991 |
| 96 | L | 93 | 99 | KSMLSYG | 1.109 |
| 97 | S | 94 | 100 | SMLSYGN | 1.187 |
| 98 | Y | 95 | 101 | MLSYGNV | 1.054 |
| 99 | G | 96 | 102 | LSYGNVI | 1.036 |
| 100 | N | 97 | 103 | SYGNVID | 1.16 |
| 101 | V | 98 | 104 | YGNVIDL | 1.04 |
| 102 | I | 99 | 105 | GNVIDLN | 1.1 |
| 103 | D | 100 | 106 | NVIDLNH | 1.013 |
| 104 | L | 101 | 107 | VIDLNHL | 0.874 |
| 105 | N | 102 | 108 | IDLNHLD | 1.011 |
| 106 | H | 103 | 109 | DLNHLDI | 1.011 |
| 107 | L | 104 | 110 | LNHLDID | 1.011 |
| 108 | D | 105 | 111 | NHLDIDE | 1.033 |
| 109 | I | 106 | 112 | HLDIDEP | 1.027 |
| 110 | D | 107 | 113 | LDIDEPT | 1.029 |
| 111 | E | 108 | 114 | DIDEPTG | 1.167 |
| 112 | P | 109 | 115 | IDEPTGQ | 1.099 |
| 113 | T | 110 | 116 | DEPTGQT | 1.169 |
| 114 | G | 111 | 117 | EPTGQTA | 1.054 |
| 115 | Q | 112 | 118 | PTGQTAD | 1.157 |
| 116 | T | 113 | 119 | TGQTADW | 1.077 |
| 117 | A | 114 | 120 | GQTADWL | 1.024 |
| 118 | D | 115 | 121 | QTADWLS | 1.006 |
| 119 | W | 116 | 122 | TADWLSI | 0.933 |
| 120 | L | 117 | 123 | ADWLSIV | 0.867 |
| 121 | S | 118 | 124 | DWLSIVV | 0.844 |
| 122 | I | 119 | 125 | WLSIVVY | 0.799 |
| 123 | V | 120 | 126 | LSIVVYL | 0.746 |
| 124 | V | 121 | 127 | SIVVYLT | 0.799 |
| 125 | Y | 122 | 128 | IVVYLTS | 0.799 |
| 126 | L | 123 | 129 | VVYLTSF | 0.817 |
| 127 | T | 124 | 130 | VYLTSFV | 0.817 |
| 128 | S | 125 | 131 | YLTSFVV | 0.817 |
| 129 | F | 126 | 132 | LTSFVVP | 0.871 |
| 130 | V | 127 | 133 | TSFVVPI | 0.854 |
| 131 | V | 128 | 134 | SFVVPIL | 0.801 |
| 132 | P | 129 | 135 | FVVPILL | 0.681 |
| 133 | I | 130 | 136 | VVPILLK | 0.74 |
| 134 | L | 131 | 137 | VPILLKA | 0.763 |
| 135 | L | 132 | 138 | PILLKAL | 0.776 |
| 136 | K | 133 | 139 | ILLKALY | 0.721 |
| 137 | A | 134 | 140 | LLKALYM | 0.74 |
| 138 | L | 135 | 141 | LKALYML | 0.74 |
| 139 | Y | 136 | 142 | KALYMLT | 0.793 |
| 140 | M | 137 | 143 | ALYMLTT | 0.786 |
| 141 | L | 138 | 144 | LYMLTTR | 0.827 |
| 142 | T | 139 | 145 | YMLTTRG | 0.966 |
| 143 | T | 140 | 146 | MLTTRGR | 0.939 |
| 144 | R | 141 | 147 | LTTRGRQ | 0.993 |
| 145 | G | 142 | 148 | TTRGRQT | 1.046 |
| 146 | R | 143 | 149 | TRGRQTT | 1.046 |
| 147 | Q | 144 | 150 | RGRQTTK | 1.053 |
| 148 | T | 145 | 151 | GRQTTKD | 1.126 |
| 149 | T | 146 | 152 | RQTTKDN | 1.126 |
| 150 | K | 147 | 153 | QTTKDNK | 1.134 |
| 151 | D | 148 | 154 | TTKDNKG | 1.217 |
| 152 | N | 149 | 155 | TKDNKGM | 1.166 |
| 153 | K | 150 | 156 | KDNKGMR | 1.164 |
| 154 | G | 151 | 157 | DNKGMRI | 1.087 |
| 155 | M | 152 | 158 | NKGMRIR | 1.014 |
| 156 | R | 153 | 159 | KGMRIRF | 0.877 |
| 157 | I | 154 | 160 | GMRIRFK | 0.877 |
| 158 | R | 155 | 161 | MRIRFKD | 0.863 |
| 159 | F | 156 | 162 | RIRFKDD | 0.986 |
| 160 | K | 157 | 163 | IRFKDDS | 1.054 |
| 161 | D | 158 | 164 | RFKDDSS | 1.191 |
| 162 | D | 159 | 165 | FKDDSSF | 1.141 |
| 163 | S | 160 | 166 | KDDSSFE | 1.161 |
| 164 | S | 161 | 167 | DDSSFED | 1.226 |
| 165 | F | 162 | 168 | DSSFEDV | 1.089 |
| 166 | E | 163 | 169 | SSFEDVN | 1.103 |
| 167 | D | 164 | 170 | SFEDVNG | 1.121 |
| 168 | V | 165 | 171 | FEDVNGI | 0.984 |
| 169 | N | 166 | 172 | EDVNGIR | 1.034 |
| 170 | G | 167 | 173 | DVNGIRK | 1.073 |
| 171 | I | 168 | 174 | VNGIRKP | 1.081 |
| 172 | R | 169 | 175 | NGIRKPK | 1.154 |
| 173 | K | 170 | 176 | GIRKPKH | 1.067 |
| 174 | P | 171 | 177 | IRKPKHL | 0.929 |
| 175 | K | 172 | 178 | RKPKHLF | 0.947 |
| 176 | H | 173 | 179 | KPKHLFL | 0.896 |
| 177 | L | 174 | 180 | PKHLFLS | 0.956 |
| 178 | F | 175 | 181 | KHLFLSM | 0.824 |
| 179 | L | 176 | 182 | HLFLSMP | 0.897 |
| 180 | S | 177 | 183 | LFLSMPN | 0.984 |
| 181 | M | 178 | 184 | FLSMPNA | 0.994 |
| 182 | P | 179 | 185 | LSMPNAQ | 1.049 |
| 183 | N | 180 | 186 | SMPNAQS | 1.169 |
| 184 | A | 181 | 187 | MPNAQSS | 1.169 |
| 185 | Q | 182 | 188 | PNAQSSM | 1.169 |
| 186 | S | 183 | 189 | NAQSSMK | 1.096 |
| 187 | S | 184 | 190 | AQSSMKA | 0.967 |
| 188 | M | 185 | 191 | QSSMKAD | 1.081 |
| 189 | K | 186 | 192 | SSMKADE | 1.047 |
| 190 | A | 187 | 193 | SMKADEI | 0.91 |
| 191 | D | 188 | 194 | MKADEIT | 0.843 |
| 192 | E | 189 | 195 | KADEITP | 0.974 |
| 193 | I | 190 | 196 | ADEITPG | 1.053 |
| 194 | T | 191 | 197 | DEITPGR | 1.094 |
| 195 | P | 192 | 198 | EITPGRF | 0.971 |
| 196 | G | 193 | 199 | ITPGRFR | 1.001 |
| 197 | R | 194 | 200 | TPGRFRT | 1.071 |
| 198 | F | 195 | 201 | PGRFRTA | 1.029 |
| 199 | R | 196 | 202 | GRFRTAI | 0.879 |
| 200 | T | 197 | 203 | RFRTAIC | 0.826 |
| 201 | A | 198 | 204 | FRTAICG | 0.913 |
| 202 | I | 199 | 205 | RTAICGL | 0.911 |
| 203 | C | 200 | 206 | TAICGLY | 0.939 |
| 204 | G | 201 | 207 | AICGLYP | 1.019 |
| 205 | L | 202 | 208 | ICGLYPA | 1.019 |
| 206 | Y | 203 | 209 | CGLYPAQ | 1.091 |
| 207 | P | 204 | 210 | GLYPAQV | 0.993 |
| 208 | A | 205 | 211 | LYPAQVK | 0.914 |
| 209 | Q | 206 | 212 | YPAQVKA | 0.924 |
| 210 | V | 207 | 213 | PAQVKAR | 0.897 |
| 211 | K | 208 | 214 | AQVKARN | 0.903 |
| 212 | A | 209 | 215 | QVKARNL | 0.893 |
| 213 | R | 210 | 216 | VKARNLI | 0.82 |
| 214 | N | 211 | 217 | KARNLIS | 0.953 |
| 215 | L | 212 | 218 | ARNLISP | 1.026 |
| 216 | I | 213 | 219 | RNLISPV | 1.003 |
| 217 | S | 214 | 220 | NLISPVM | 0.953 |
| 218 | P | 215 | 221 | LISPVMS | 0.934 |
| 219 | V | 216 | 222 | ISPVMSV | 0.921 |
| 220 | M | 217 | 223 | SPVMSVI | 0.921 |
| 221 | S | 218 | 224 | PVMSVIG | 0.94 |
| 222 | V | 219 | 225 | VMSVIGF | 0.809 |
| 223 | I | 220 | 226 | MSVIGFL | 0.821 |
| 224 | G | 221 | 227 | SVIGFLA | 0.83 |
| 225 | F | 222 | 228 | VIGFLAL | 0.71 |
| 226 | L | 223 | 229 | IGFLALA | 0.733 |
| 227 | A | 224 | 230 | GFLALAK | 0.81 |
| 228 | L | 225 | 231 | FLALAKN | 0.81 |
| 229 | A | 226 | 232 | LALAKNW | 0.861 |
| 230 | K | 227 | 233 | ALAKNWT | 0.914 |
| 231 | N | 228 | 234 | LAKNWTE | 0.926 |
| 232 | W | 229 | 235 | AKNWTER | 0.977 |
| 233 | T | 230 | 236 | KNWTERV | 0.954 |
| 234 | E | 231 | 237 | NWTERVE | 0.916 |
| 235 | R | 232 | 238 | WTERVEE | 0.799 |
| 236 | V | 233 | 239 | TERVEEW | 0.799 |
| 237 | E | 234 | 240 | ERVEEWL | 0.746 |
| 238 | E | 235 | 241 | RVEEWLD | 0.849 |
| 239 | W | 236 | 242 | VEEWLDL | 0.797 |
| 240 | L | 237 | 243 | EEWLDLP | 0.943 |
| 241 | D | 238 | 244 | EWLDLPC | 1.007 |
| 242 | L | 239 | 245 | WLDLPCK | 1.046 |
| 243 | P | 240 | 246 | LDLPCKL | 0.993 |
| 244 | C | 241 | 247 | DLPCKLL | 0.993 |
| 245 | K | 242 | 248 | LPCKLLS | 0.989 |
| 246 | L | 243 | 249 | PCKLLSE | 1.01 |
| 247 | L | 244 | 250 | CKLLSEP | 1.01 |
| 248 | S | 245 | 251 | KLLSEPS | 1.044 |
| 249 | E | 246 | 252 | LLSEPSP | 1.117 |
| 250 | P | 247 | 253 | LSEPSPT | 1.17 |
| 251 | S | 248 | 254 | SEPSPTS | 1.29 |
| 252 | P | 249 | 255 | EPSPTSL | 1.17 |
| 253 | T | 250 | 256 | PSPTSLT | 1.201 |
| 254 | S | 251 | 257 | SPTSLTK | 1.129 |
| 255 | L | 252 | 258 | PTSLTKG | 1.147 |
| 256 | T | 253 | 259 | TSLTKGP | 1.147 |
| 257 | K | 254 | 260 | SLTKGPS | 1.214 |
| 258 | G | 255 | 261 | LTKGPST | 1.147 |
| 259 | P | 256 | 262 | TKGPSTN | 1.286 |
| 260 | S | 257 | 263 | KGPSTNR | 1.284 |
| 261 | T | 258 | 264 | GPSTNRD | 1.349 |
| 262 | N | 259 | 265 | PSTNRDY | 1.289 |
| 263 | R | 260 | 266 | STNRDYL | 1.156 |
| 264 | D | 261 | 267 | TNRDYLN | 1.174 |
| 265 | Y | 262 | 268 | NRDYLNQ | 1.177 |
| 266 | L | 263 | 269 | RDYLNQR | 1.09 |
| 267 | N | 264 | 270 | DYLNQRQ | 1.094 |
| 268 | Q | 265 | 271 | YLNQRQG | 1.109 |
| 269 | R | 266 | 272 | LNQRQGA | 1.04 |
| 270 | Q | 267 | 273 | NQRQGAL | 1.04 |
| 271 | G | 268 | 274 | QRQGALA | 0.911 |
| 272 | A | 269 | 275 | RQGALAK | 0.916 |
| 273 | L | 270 | 276 | QGALAKM | 0.866 |
| 274 | A | 271 | 277 | GALAKME | 0.831 |
| 275 | K | 272 | 278 | ALAKMET | 0.746 |
| 276 | M | 273 | 279 | LAKMETK | 0.796 |
| 277 | E | 274 | 280 | AKMETKE | 0.817 |
| 278 | T | 275 | 281 | KMETKEA | 0.817 |
| 279 | K | 276 | 282 | METKEAQ | 0.813 |
| 280 | E | 277 | 283 | ETKEAQA | 0.821 |
| 281 | A | 278 | 284 | TKEAQAV | 0.787 |
| 282 | Q | 279 | 285 | KEAQAVR | 0.786 |
| 283 | A | 280 | 286 | EAQAVRK | 0.786 |
| 284 | V | 281 | 287 | AQAVRKH | 0.816 |
| 285 | R | 282 | 288 | QAVRKHA | 0.816 |
| 286 | K | 283 | 289 | AVRKHAI | 0.743 |
| 287 | H | 284 | 290 | VRKHAID | 0.857 |
| 288 | A | 285 | 291 | RKHAIDA | 0.88 |
| 289 | I | 286 | 292 | KHAIDAG | 0.967 |
| 290 | D | 287 | 293 | HAIDAGC | 0.993 |
| 291 | A | 288 | 294 | AIDAGCN | 1.08 |
| 292 | G | 289 | 295 | IDAGCNL | 1.07 |
| 293 | C | 290 | 296 | DAGCNLI | 1.07 |
| 294 | N | 291 | 297 | AGCNLID | 1.07 |
| 295 | L | 292 | 298 | GCNLIDH | 1.111 |
| 296 | I | 293 | 299 | CNLIDHI | 0.956 |
| 297 | D | 294 | 300 | NLIDHID | 0.994 |
| 298 | H | 295 | 301 | LIDHIDS | 0.976 |
| 299 | I | 296 | 302 | IDHIDSP | 1.109 |
| 300 | D | 297 | 303 | DHIDSPS | 1.246 |
| 301 | S | 298 | 304 | HIDSPSS | 1.241 |
| 302 | P | 299 | 305 | IDSPSSI | 1.173 |
| 303 | S | 300 | 306 | DSPSSIW | 1.243 |
| 304 | S | 301 | 307 | SPSSIWV | 1.106 |
| 305 | I | 302 | 308 | PSSIWVF | 0.987 |
| 306 | W | 303 | 309 | SSIWVFA | 0.864 |
| 307 | V | 304 | 310 | SIWVFAG | 0.883 |
| 308 | F | 305 | 311 | IWVFAGA | 0.773 |
| 309 | A | 306 | 312 | WVFAGAP | 0.923 |
| 310 | G | 307 | 313 | VFAGAPD | 0.994 |
| 311 | A | 308 | 314 | FAGAPDR | 1.059 |
| 312 | P | 309 | 315 | AGAPDRC | 1.143 |
| 313 | D | 310 | 316 | GAPDRCP | 1.266 |
| 314 | R | 311 | 317 | APDRCPP | 1.26 |
| 315 | C | 312 | 318 | PDRCPPT | 1.303 |
| 316 | P | 313 | 319 | DRCPPTC | 1.256 |
| 317 | P | 314 | 320 | RCPPTCL | 1.131 |
| 318 | T | 315 | 321 | CPPTCLF | 1.081 |
| 319 | C | 316 | 322 | PPTCLFI | 0.979 |
| 320 | L | 317 | 323 | PTCLFIA | 0.856 |
| 321 | F | 318 | 324 | TCLFIAG | 0.861 |
| 322 | I | 319 | 325 | CLFIAGM | 0.81 |
| 323 | A | 320 | 326 | LFIAGMA | 0.734 |
| 324 | G | 321 | 327 | FIAGMAE | 0.756 |
| 325 | M | 322 | 328 | IAGMAEL | 0.754 |
| 326 | A | 323 | 329 | AGMAELG | 0.91 |
| 327 | E | 324 | 330 | GMAELGA | 0.91 |
| 328 | L | 325 | 331 | MAELGAF | 0.773 |
| 329 | G | 326 | 332 | AELGAFF | 0.773 |
| 330 | A | 327 | 333 | ELGAFFA | 0.773 |
| 331 | F | 328 | 334 | LGAFFAV | 0.739 |
| 332 | F | 329 | 335 | GAFFAVL | 0.739 |
| 333 | A | 330 | 336 | AFFAVLQ | 0.656 |
| 334 | V | 331 | 337 | FFAVLQD | 0.77 |
| 335 | L | 332 | 338 | FAVLQDM | 0.77 |
| 336 | Q | 333 | 339 | AVLQDMR | 0.82 |
| 337 | D | 334 | 340 | VLQDMRN | 0.949 |
| 338 | M | 335 | 341 | LQDMRNT | 1.014 |
| 339 | R | 336 | 342 | QDMRNTI | 0.997 |
| 340 | N | 337 | 343 | DMRNTIM | 0.943 |
| 341 | T | 338 | 344 | MRNTIMA | 0.829 |
| 342 | I | 339 | 345 | RNTIMAS | 0.947 |
| 343 | M | 340 | 346 | NTIMASK | 0.956 |
| 344 | A | 341 | 347 | TIMASKT | 0.87 |
| 345 | S | 342 | 348 | IMASKTI | 0.8 |
| 346 | K | 343 | 349 | MASKTIG | 0.956 |
| 347 | T | 344 | 350 | ASKTIGT | 1.007 |
| 348 | I | 345 | 351 | SKTIGTS | 1.117 |
| 349 | G | 346 | 352 | KTIGTSE | 1.019 |
| 350 | T | 347 | 353 | TIGTSEE | 0.98 |
| 351 | S | 348 | 354 | IGTSEEK | 0.987 |
| 352 | E | 349 | 355 | GTSEEKL | 1.004 |
| 353 | E | 350 | 356 | TSEEKLK | 0.926 |
| 354 | K | 351 | 357 | SEEKLKK | 0.933 |
| 355 | L | 352 | 358 | EEKLKKK | 0.873 |
| 356 | K | 353 | 359 | EKLKKKS | 0.971 |
| 357 | K | 354 | 360 | KLKKKSS | 1.07 |
| 358 | K | 355 | 361 | LKKKSSF | 1.011 |
| 359 | S | 356 | 362 | KKKSSFY | 1.09 |
| 360 | S | 357 | 363 | KKSSFYQ | 1.086 |
| 361 | F | 358 | 364 | KSSFYQS | 1.146 |
| 362 | Y | 359 | 365 | SSFYQSY | 1.164 |
| 363 | Q | 360 | 366 | SFYQSYL | 1.044 |
| 364 | S | 361 | 367 | FYQSYLR | 0.976 |
| 365 | Y | 362 | 368 | YQSYLRR | 1.026 |
| 366 | L | 363 | 369 | QSYLRRT | 1 |
| 367 | R | 364 | 370 | SYLRRTQ | 1 |
| 368 | R | 365 | 371 | YLRRTQS | 1 |
| 369 | T | 366 | 372 | LRRTQSM | 0.923 |
| 370 | Q | 367 | 373 | RRTQSMG | 1.061 |
| 371 | S | 368 | 374 | RTQSMGI | 0.993 |
| 372 | M | 369 | 375 | TQSMGIQ | 0.997 |
| 373 | G | 370 | 376 | QSMGIQL | 0.944 |
| 374 | I | 371 | 377 | SMGIQLD | 1.013 |
| 375 | Q | 372 | 378 | MGIQLDQ | 0.949 |
| 376 | L | 373 | 379 | GIQLDQR | 0.999 |
| 377 | D | 374 | 380 | IQLDQRI | 0.843 |
| 378 | Q | 375 | 381 | QLDQRII | 0.843 |
| 379 | R | 376 | 382 | LDQRIIV | 0.774 |
| 380 | I | 377 | 383 | DQRIIVL | 0.774 |
| 381 | I | 378 | 384 | QRIIVLF | 0.651 |
| 382 | V | 379 | 385 | RIIVLFM | 0.597 |
| 383 | L | 380 | 386 | IIVLFMV | 0.533 |
| 384 | F | 381 | 387 | IVLFMVD | 0.674 |
| 385 | M | 382 | 388 | VLFMVDW | 0.744 |
| 386 | V | 383 | 389 | LFMVDWG | 0.896 |
| 387 | D | 384 | 390 | FMVDWGK | 0.956 |
| 388 | W | 385 | 391 | MVDWGKE | 0.976 |
| 389 | G | 386 | 392 | VDWGKEA | 0.984 |
| 390 | K | 387 | 393 | DWGKEAV | 0.984 |
| 391 | E | 388 | 394 | WGKEAVD | 0.984 |
| 392 | A | 389 | 395 | GKEAVDS | 1.051 |
| 393 | V | 390 | 396 | KEAVDSF | 0.914 |
| 394 | D | 391 | 397 | EAVDSFH | 0.906 |
| 395 | S | 392 | 398 | AVDSFHL | 0.884 |
| 396 | F | 393 | 399 | VDSFHLG | 1.013 |
| 397 | H | 394 | 400 | DSFHLGD | 1.15 |
| 398 | L | 395 | 401 | SFHLGDD | 1.15 |
| 399 | G | 396 | 402 | FHLGDDM | 1.031 |
| 400 | D | 397 | 403 | HLGDDMD | 1.154 |
| 401 | D | 398 | 404 | LGDDMDP | 1.236 |
| 402 | M | 399 | 405 | GDDMDPE | 1.257 |
| 403 | D | 400 | 406 | DDMDPEL | 1.119 |
| 404 | P | 401 | 407 | DMDPELR | 1.046 |
| 405 | E | 402 | 408 | MDPELRG | 1.06 |
| 406 | L | 403 | 409 | DPELRGL | 1.059 |
| 407 | R | 404 | 410 | PELRGLA | 0.944 |
| 408 | G | 405 | 411 | ELRGLAQ | 0.867 |
| 409 | L | 406 | 412 | LRGLAQA | 0.856 |
| 410 | A | 407 | 413 | RGLAQAL | 0.856 |
| 411 | Q | 408 | 414 | GLAQALI | 0.787 |
| 412 | A | 409 | 415 | LAQALID | 0.773 |
| 413 | L | 410 | 416 | AQALIDQ | 0.829 |
| 414 | I | 411 | 417 | QALIDQK | 0.879 |
| 415 | D | 412 | 418 | ALIDQKV | 0.81 |
| 416 | Q | 413 | 419 | LIDQKVK | 0.86 |
| 417 | K | 414 | 420 | IDQKVKE | 0.881 |
| 418 | V | 415 | 421 | DQKVKEI | 0.881 |
| 419 | K | 416 | 422 | QKVKEIS | 0.877 |
| 420 | E | 417 | 423 | KVKEISN | 0.96 |
| 421 | I | 418 | 424 | VKEISNQ | 0.956 |
| 422 | S | 419 | 425 | KEISNQE | 0.99 |
| 423 | N | 420 | 426 | EISNQEP | 1.063 |
| 424 | Q | 421 | 427 | ISNQEPL | 1.041 |
| 425 | E | 422 | 428 | SNQEPLK | 1.119 |
| 426 | P | 423 | 429 | NQEPLKL | 0.999 |
| **Hantaan Virus** | | |  | | |
| 4 | M | 1 | 7 | MATMEEL | 0.699 |
| 5 | E | 2 | 8 | ATMEELQ | 0.753 |
| 6 | E | 3 | 9 | TMEELQR | 0.794 |
| 7 | L | 4 | 10 | MEELQRE | 0.763 |
| 8 | Q | 5 | 11 | EELQREI | 0.744 |
| 9 | R | 6 | 12 | ELQREIN | 0.861 |
| 10 | E | 7 | 13 | LQREINA | 0.85 |
| 11 | I | 8 | 14 | QREINAH | 0.901 |
| 12 | N | 9 | 15 | REINAHE | 0.867 |
| 13 | A | 10 | 16 | EINAHEG | 0.954 |
| 14 | H | 11 | 17 | INAHEGQ | 0.989 |
| 15 | E | 12 | 18 | NAHEGQL | 1.006 |
| 16 | G | 13 | 19 | AHEGQLV | 0.854 |
| 17 | Q | 14 | 20 | HEGQLVI | 0.827 |
| 18 | L | 15 | 21 | EGQLVIA | 0.786 |
| 19 | V | 16 | 22 | GQLVIAR | 0.816 |
| 20 | I | 17 | 23 | QLVIARQ | 0.733 |
| 21 | A | 18 | 24 | LVIARQK | 0.737 |
| 22 | R | 19 | 25 | VIARQKV | 0.724 |
| 23 | Q | 20 | 26 | IARQKVR | 0.789 |
| 24 | K | 21 | 27 | ARQKVRD | 0.93 |
| 25 | V | 22 | 28 | RQKVRDA | 0.93 |
| 26 | R | 23 | 29 | QKVRDAE | 0.9 |
| 27 | D | 24 | 30 | KVRDAEK | 0.904 |
| 28 | A | 25 | 31 | VRDAEKQ | 0.9 |
| 29 | E | 26 | 32 | RDAEKQY | 0.991 |
| 30 | K | 27 | 33 | DAEKQYE | 0.961 |
| 31 | Q | 28 | 34 | AEKQYEK | 0.897 |
| 32 | Y | 29 | 35 | EKQYEKD | 1.011 |
| 33 | E | 30 | 36 | KQYEKDP | 1.123 |
| 34 | K | 31 | 37 | QYEKDPD | 1.187 |
| 35 | D | 32 | 38 | YEKDPDE | 1.153 |
| 36 | P | 33 | 39 | EKDPDEL | 1.074 |
| 37 | D | 34 | 40 | KDPDELN | 1.191 |
| 38 | E | 35 | 41 | DPDELNK | 1.191 |
| 39 | L | 36 | 42 | PDELNKR | 1.119 |
| 40 | N | 37 | 43 | DELNKRT | 1.039 |
| 41 | K | 38 | 44 | ELNKRTL | 0.914 |
| 42 | R | 39 | 45 | LNKRTLT | 0.946 |
| 43 | T | 40 | 46 | NKRTLTD | 1.07 |
| 44 | L | 41 | 47 | KRTLTDR | 0.983 |
| 45 | T | 42 | 48 | RTLTDRE | 0.944 |
| 46 | D | 43 | 49 | TLTDREG | 1.031 |
| 47 | R | 44 | 50 | LTDREGV | 0.966 |
| 48 | E | 45 | 51 | TDREGVA | 0.976 |
| 49 | G | 46 | 52 | DREGVAV | 0.91 |
| 50 | V | 47 | 53 | REGVAVS | 0.906 |
| 51 | A | 48 | 54 | EGVAVSI | 0.837 |
| 52 | V | 49 | 55 | GVAVSIQ | 0.871 |
| 53 | S | 50 | 56 | VAVSIQA | 0.743 |
| 54 | I | 51 | 57 | AVSIQAK | 0.816 |
| 55 | Q | 52 | 58 | VSIQAKI | 0.789 |
| 56 | A | 53 | 59 | SIQAKID | 0.926 |
| 57 | K | 54 | 60 | IQAKIDE | 0.827 |
| 58 | I | 55 | 61 | QAKIDEL | 0.844 |
| 59 | D | 56 | 62 | AKIDELK | 0.849 |
| 60 | E | 57 | 63 | KIDELKR | 0.89 |
| 61 | L | 58 | 64 | IDELKRQ | 0.886 |
| 62 | K | 59 | 65 | DELKRQL | 0.903 |
| 63 | R | 60 | 66 | ELKRQLA | 0.789 |
| 64 | Q | 61 | 67 | LKRQLAD | 0.891 |
| 65 | L | 62 | 68 | KRQLADR | 0.943 |
| 66 | A | 63 | 69 | RQLADRI | 0.866 |
| 67 | D | 64 | 70 | QLADRIA | 0.824 |
| 68 | R | 65 | 71 | LADRIAT | 0.821 |
| 69 | I | 66 | 72 | ADRIATG | 0.96 |
| 70 | A | 67 | 73 | DRIATGK | 1.01 |
| 71 | T | 68 | 74 | RIATGKN | 1.024 |
| 72 | G | 69 | 75 | IATGKNL | 0.973 |
| 73 | K | 70 | 76 | ATGKNLG | 1.129 |
| 74 | N | 71 | 77 | TGKNLGK | 1.179 |
| 75 | L | 72 | 78 | GKNLGKE | 1.147 |
| 76 | G | 73 | 79 | KNLGKEQ | 1.064 |
| 77 | K | 74 | 80 | NLGKEQD | 1.129 |
| 78 | E | 75 | 81 | LGKEQDP | 1.123 |
| 79 | Q | 76 | 82 | GKEQDPT | 1.176 |
| 80 | D | 77 | 83 | KEQDPTG | 1.176 |
| 81 | P | 78 | 84 | EQDPTGV | 1.103 |
| 82 | T | 79 | 85 | QDPTGVE | 1.103 |
| 83 | G | 80 | 86 | DPTGVEP | 1.18 |
| 84 | V | 81 | 87 | PTGVEPG | 1.194 |
| 85 | E | 82 | 88 | TGVEPGD | 1.186 |
| 86 | P | 83 | 89 | GVEPGDH | 1.184 |
| 87 | G | 84 | 90 | VEPGDHL | 1.046 |
| 88 | D | 85 | 91 | EPGDHLK | 1.119 |
| 89 | H | 86 | 92 | PGDHLKE | 1.119 |
| 90 | L | 87 | 93 | GDHLKER | 1.037 |
| 91 | K | 88 | 94 | DHLKERS | 1.019 |
| 92 | E | 89 | 95 | HLKERSM | 0.896 |
| 93 | R | 90 | 96 | LKERSML | 0.844 |
| 94 | S | 91 | 97 | KERSMLS | 0.964 |
| 95 | M | 92 | 98 | ERSMLSY | 0.983 |
| 96 | L | 93 | 99 | RSMLSYG | 1.1 |
| 97 | S | 94 | 100 | SMLSYGN | 1.187 |
| 98 | Y | 95 | 101 | MLSYGNV | 1.054 |
| 99 | G | 96 | 102 | LSYGNVL | 1.053 |
| 100 | N | 97 | 103 | SYGNVLD | 1.177 |
| 101 | V | 98 | 104 | YGNVLDL | 1.057 |
| 102 | L | 99 | 105 | GNVLDLN | 1.117 |
| 103 | D | 100 | 106 | NVLDLNH | 1.03 |
| 104 | L | 101 | 107 | VLDLNHL | 0.891 |
| 105 | N | 102 | 108 | LDLNHLD | 1.029 |
| 106 | H | 103 | 109 | DLNHLDI | 1.011 |
| 107 | L | 104 | 110 | LNHLDID | 1.011 |
| 108 | D | 105 | 111 | NHLDIDE | 1.033 |
| 109 | I | 106 | 112 | HLDIDEP | 1.027 |
| 110 | D | 107 | 113 | LDIDEPT | 1.029 |
| 111 | E | 108 | 114 | DIDEPTG | 1.167 |
| 112 | P | 109 | 115 | IDEPTGQ | 1.099 |
| 113 | T | 110 | 116 | DEPTGQT | 1.169 |
| 114 | G | 111 | 117 | EPTGQTA | 1.054 |
| 115 | Q | 112 | 118 | PTGQTAD | 1.157 |
| 116 | T | 113 | 119 | TGQTADW | 1.077 |
| 117 | A | 114 | 120 | GQTADWL | 1.024 |
| 118 | D | 115 | 121 | QTADWLS | 1.006 |
| 119 | W | 116 | 122 | TADWLSI | 0.933 |
| 120 | L | 117 | 123 | ADWLSII | 0.863 |
| 121 | S | 118 | 124 | DWLSIIV | 0.84 |
| 122 | I | 119 | 125 | WLSIIVY | 0.794 |
| 123 | I | 120 | 126 | LSIIVYL | 0.741 |
| 124 | V | 121 | 127 | SIIVYLT | 0.794 |
| 125 | Y | 122 | 128 | IIVYLTS | 0.794 |
| 126 | L | 123 | 129 | IVYLTSF | 0.813 |
| 127 | T | 124 | 130 | VYLTSFV | 0.817 |
| 128 | S | 125 | 131 | YLTSFVV | 0.817 |
| 129 | F | 126 | 132 | LTSFVVP | 0.871 |
| 130 | V | 127 | 133 | TSFVVPI | 0.854 |
| 131 | V | 128 | 134 | SFVVPIL | 0.801 |
| 132 | P | 129 | 135 | FVVPILL | 0.681 |
| 133 | I | 130 | 136 | VVPILLK | 0.74 |
| 134 | L | 131 | 137 | VPILLKA | 0.763 |
| 135 | L | 132 | 138 | PILLKAL | 0.776 |
| 136 | K | 133 | 139 | ILLKALY | 0.721 |
| 137 | A | 134 | 140 | LLKALYM | 0.74 |
| 138 | L | 135 | 141 | LKALYML | 0.74 |
| 139 | Y | 136 | 142 | KALYMLT | 0.793 |
| 140 | M | 137 | 143 | ALYMLTT | 0.786 |
| 141 | L | 138 | 144 | LYMLTTR | 0.827 |
| 142 | T | 139 | 145 | YMLTTRG | 0.966 |
| 143 | T | 140 | 146 | MLTTRGR | 0.939 |
| 144 | R | 141 | 147 | LTTRGRQ | 0.993 |
| 145 | G | 142 | 148 | TTRGRQT | 1.046 |
| 146 | R | 143 | 149 | TRGRQTT | 1.046 |
| 147 | Q | 144 | 150 | RGRQTTK | 1.053 |
| 148 | T | 145 | 151 | GRQTTKD | 1.126 |
| 149 | T | 146 | 152 | RQTTKDN | 1.126 |
| 150 | K | 147 | 153 | QTTKDNK | 1.134 |
| 151 | D | 148 | 154 | TTKDNKG | 1.217 |
| 152 | N | 149 | 155 | TKDNKGT | 1.217 |
| 153 | K | 150 | 156 | KDNKGTR | 1.216 |
| 154 | G | 151 | 157 | DNKGTRI | 1.139 |
| 155 | T | 152 | 158 | NKGTRIR | 1.066 |
| 156 | R | 153 | 159 | KGTRIRF | 0.929 |
| 157 | I | 154 | 160 | GTRIRFK | 0.929 |
| 158 | R | 155 | 161 | TRIRFKD | 0.914 |
| 159 | F | 156 | 162 | RIRFKDD | 0.986 |
| 160 | K | 157 | 163 | IRFKDDS | 1.054 |
| 161 | D | 158 | 164 | RFKDDSS | 1.191 |
| 162 | D | 159 | 165 | FKDDSSF | 1.141 |
| 163 | S | 160 | 166 | KDDSSFE | 1.161 |
| 164 | S | 161 | 167 | DDSSFED | 1.226 |
| 165 | F | 162 | 168 | DSSFEDV | 1.089 |
| 166 | E | 163 | 169 | SSFEDVN | 1.103 |
| 167 | D | 164 | 170 | SFEDVNG | 1.121 |
| 168 | V | 165 | 171 | FEDVNGI | 0.984 |
| 169 | N | 166 | 172 | EDVNGIR | 1.034 |
| 170 | G | 167 | 173 | DVNGIRK | 1.073 |
| 171 | I | 168 | 174 | VNGIRKP | 1.081 |
| 172 | R | 169 | 175 | NGIRKPK | 1.154 |
| 173 | K | 170 | 176 | GIRKPKH | 1.067 |
| 174 | P | 171 | 177 | IRKPKHL | 0.929 |
| 175 | K | 172 | 178 | RKPKHLY | 1.024 |
| 176 | H | 173 | 179 | KPKHLYV | 0.96 |
| 177 | L | 174 | 180 | PKHLYVS | 1.02 |
| 178 | Y | 175 | 181 | KHLYVSL | 0.887 |
| 179 | V | 176 | 182 | HLYVSLP | 0.96 |
| 180 | S | 177 | 183 | LYVSLPN | 1.047 |
| 181 | L | 178 | 184 | YVSLPNA | 1.057 |
| 182 | P | 179 | 185 | VSLPNAQ | 1.034 |
| 183 | N | 180 | 186 | SLPNAQS | 1.167 |
| 184 | A | 181 | 187 | LPNAQSS | 1.167 |
| 185 | Q | 182 | 188 | PNAQSSM | 1.169 |
| 186 | S | 183 | 189 | NAQSSMK | 1.096 |
| 187 | S | 184 | 190 | AQSSMKA | 0.967 |
| 188 | M | 185 | 191 | QSSMKAE | 0.979 |
| 189 | K | 186 | 192 | SSMKAEE | 0.944 |
| 190 | A | 187 | 193 | SMKAEEI | 0.807 |
| 191 | E | 188 | 194 | MKAEEIT | 0.74 |
| 192 | E | 189 | 195 | KAEEITP | 0.871 |
| 193 | I | 190 | 196 | AEEITPG | 0.95 |
| 194 | T | 191 | 197 | EEITPGR | 0.991 |
| 195 | P | 192 | 198 | EITPGRY | 1.049 |
| 196 | G | 193 | 199 | ITPGRYR | 1.079 |
| 197 | R | 194 | 200 | TPGRYRT | 1.149 |
| 198 | Y | 195 | 201 | PGRYRTA | 1.106 |
| 199 | R | 196 | 202 | GRYRTAV | 0.96 |
| 200 | T | 197 | 203 | RYRTAVC | 0.907 |
| 201 | A | 198 | 204 | YRTAVCG | 0.994 |
| 202 | V | 199 | 205 | RTAVCGL | 0.916 |
| 203 | C | 200 | 206 | TAVCGLY | 0.943 |
| 204 | G | 201 | 207 | AVCGLYP | 1.023 |
| 205 | L | 202 | 208 | VCGLYPA | 1.023 |
| 206 | Y | 203 | 209 | CGLYPAQ | 1.091 |
| 207 | P | 204 | 210 | GLYPAQI | 0.989 |
| 208 | A | 205 | 211 | LYPAQIK | 0.91 |
| 209 | Q | 206 | 212 | YPAQIKA | 0.92 |
| 210 | I | 207 | 213 | PAQIKAR | 0.893 |
| 211 | K | 208 | 214 | AQIKARQ | 0.816 |
| 212 | A | 209 | 215 | QIKARQM | 0.807 |
| 213 | R | 210 | 216 | IKARQMI | 0.734 |
| 214 | Q | 211 | 217 | KARQMIS | 0.871 |
| 215 | M | 212 | 218 | ARQMISP | 0.944 |
| 216 | I | 213 | 219 | RQMISPV | 0.921 |
| 217 | S | 214 | 220 | QMISPVM | 0.871 |
| 218 | P | 215 | 221 | MISPVMS | 0.936 |
| 219 | V | 216 | 222 | ISPVMSV | 0.921 |
| 220 | M | 217 | 223 | SPVMSVI | 0.921 |
| 221 | S | 218 | 224 | PVMSVIG | 0.94 |
| 222 | V | 219 | 225 | VMSVIGF | 0.809 |
| 223 | I | 220 | 226 | MSVIGFL | 0.821 |
| 224 | G | 221 | 227 | SVIGFLA | 0.83 |
| 225 | F | 222 | 228 | VIGFLAL | 0.71 |
| 226 | L | 223 | 229 | IGFLALA | 0.733 |
| 227 | A | 224 | 230 | GFLALAK | 0.81 |
| 228 | L | 225 | 231 | FLALAKD | 0.796 |
| 229 | A | 226 | 232 | LALAKDW | 0.847 |
| 230 | K | 227 | 233 | ALAKDWS | 0.967 |
| 231 | D | 228 | 234 | LAKDWSD | 1.081 |
| 232 | W | 229 | 235 | AKDWSDR | 1.133 |
| 233 | S | 230 | 236 | KDWSDRI | 1.106 |
| 234 | D | 231 | 237 | DWSDRIE | 1.067 |
| 235 | R | 232 | 238 | WSDRIEQ | 0.999 |
| 236 | I | 233 | 239 | SDRIEQW | 0.999 |
| 237 | E | 234 | 240 | DRIEQWL | 0.879 |
| 238 | Q | 235 | 241 | RIEQWLI | 0.737 |
| 239 | W | 236 | 242 | IEQWLIE | 0.707 |
| 240 | L | 237 | 243 | EQWLIEP | 0.857 |
| 241 | I | 238 | 244 | QWLIEPC | 0.921 |
| 242 | E | 239 | 245 | WLIEPCK | 0.926 |
| 243 | P | 240 | 246 | LIEPCKL | 0.873 |
| 244 | C | 241 | 247 | IEPCKLL | 0.873 |
| 245 | K | 242 | 248 | EPCKLLP | 1.023 |
| 246 | L | 243 | 249 | PCKLLPD | 1.126 |
| 247 | L | 244 | 250 | CKLLPDT | 1.046 |
| 248 | P | 245 | 251 | KLLPDTA | 0.97 |
| 249 | D | 246 | 252 | LLPDTAA | 0.92 |
| 250 | T | 247 | 253 | LPDTAAV | 0.907 |
| 251 | A | 248 | 254 | PDTAAVS | 1.027 |
| 252 | A | 249 | 255 | DTAAVSL | 0.894 |
| 253 | V | 250 | 256 | TAAVSLL | 0.77 |
| 254 | S | 251 | 257 | AAVSLLG | 0.856 |
| 255 | L | 252 | 258 | AVSLLGG | 0.984 |
| 256 | L | 253 | 259 | VSLLGGP | 1.107 |
| 257 | G | 254 | 260 | SLLGGPA | 1.13 |
| 258 | G | 255 | 261 | LLGGPAT | 1.063 |
| 259 | P | 256 | 262 | LGGPATN | 1.201 |
| 260 | A | 257 | 263 | GGPATNR | 1.253 |
| 261 | T | 258 | 264 | GPATNRD | 1.239 |
| 262 | N | 259 | 265 | PATNRDY | 1.179 |
| 263 | R | 260 | 266 | ATNRDYL | 1.046 |
| 264 | D | 261 | 267 | TNRDYLR | 1.087 |
| 265 | Y | 262 | 268 | NRDYLRQ | 1.09 |
| 266 | L | 263 | 269 | RDYLRQR | 1.003 |
| 267 | R | 264 | 270 | DYLRQRQ | 1.007 |
| 268 | Q | 265 | 271 | YLRQRQV | 0.87 |
| 269 | R | 266 | 272 | LRQRQVA | 0.801 |
| 270 | Q | 267 | 273 | RQRQVAL | 0.801 |
| 271 | V | 268 | 274 | QRQVALG | 0.889 |
| 272 | A | 269 | 275 | RQVALGN | 0.971 |
| 273 | L | 270 | 276 | QVALGNM | 0.921 |
| 274 | G | 271 | 277 | VALGNME | 0.887 |
| 275 | N | 272 | 278 | ALGNMET | 0.953 |
| 276 | M | 273 | 279 | LGNMETK | 1.003 |
| 277 | E | 274 | 280 | GNMETKE | 1.024 |
| 278 | T | 275 | 281 | NMETKES | 1.006 |
| 279 | K | 276 | 282 | METKESK | 0.927 |
| 280 | E | 277 | 283 | ETKESKA | 0.936 |
| 281 | S | 278 | 284 | TKESKAI | 0.897 |
| 282 | K | 279 | 285 | KESKAIR | 0.896 |
| 283 | A | 280 | 286 | ESKAIRQ | 0.891 |
| 284 | I | 281 | 287 | SKAIRQH | 0.921 |
| 285 | R | 282 | 288 | KAIRQHA | 0.811 |
| 286 | Q | 283 | 289 | AIRQHAE | 0.773 |
| 287 | H | 284 | 290 | IRQHAEA | 0.773 |
| 288 | A | 285 | 291 | RQHAEAA | 0.8 |
| 289 | E | 286 | 292 | QHAEAAG | 0.887 |
| 290 | A | 287 | 293 | HAEAAGC | 0.917 |
| 291 | A | 288 | 294 | AEAAGCS | 0.986 |
| 292 | G | 289 | 295 | EAAGCSM | 0.977 |
| 293 | C | 290 | 296 | AAGCSMI | 0.939 |
| 294 | S | 291 | 297 | AGCSMIE | 0.95 |
| 295 | M | 292 | 298 | GCSMIED | 1.064 |
| 296 | I | 293 | 299 | CSMIEDI | 0.909 |
| 297 | E | 294 | 300 | SMIEDIE | 0.844 |
| 298 | D | 295 | 301 | MIEDIES | 0.844 |
| 299 | I | 296 | 302 | IEDIESP | 0.976 |
| 300 | E | 297 | 303 | EDIESPS | 1.113 |
| 301 | S | 298 | 304 | DIESPSS | 1.211 |
| 302 | P | 299 | 305 | IESPSSI | 1.07 |
| 303 | S | 300 | 306 | ESPSSIW | 1.14 |
| 304 | S | 301 | 307 | SPSSIWV | 1.106 |
| 305 | I | 302 | 308 | PSSIWVF | 0.987 |
| 306 | W | 303 | 309 | SSIWVFA | 0.864 |
| 307 | V | 304 | 310 | SIWVFAG | 0.883 |
| 308 | F | 305 | 311 | IWVFAGA | 0.773 |
| 309 | A | 306 | 312 | WVFAGAP | 0.923 |
| 310 | G | 307 | 313 | VFAGAPD | 0.994 |
| 311 | A | 308 | 314 | FAGAPDR | 1.059 |
| 312 | P | 309 | 315 | AGAPDRC | 1.143 |
| 313 | D | 310 | 316 | GAPDRCP | 1.266 |
| 314 | R | 311 | 317 | APDRCPP | 1.26 |
| 315 | C | 312 | 318 | PDRCPPT | 1.303 |
| 316 | P | 313 | 319 | DRCPPTC | 1.256 |
| 317 | P | 314 | 320 | RCPPTCL | 1.131 |
| 318 | T | 315 | 321 | CPPTCLF | 1.081 |
| 319 | C | 316 | 322 | PPTCLFI | 0.979 |
| 320 | L | 317 | 323 | PTCLFIA | 0.856 |
| 321 | F | 318 | 324 | TCLFIAG | 0.861 |
| 322 | I | 319 | 325 | CLFIAGI | 0.791 |
| 323 | A | 320 | 326 | LFIAGIA | 0.716 |
| 324 | G | 321 | 327 | FIAGIAE | 0.737 |
| 325 | I | 322 | 328 | IAGIAEL | 0.736 |
| 326 | A | 323 | 329 | AGIAELG | 0.891 |
| 327 | E | 324 | 330 | GIAELGA | 0.891 |
| 328 | L | 325 | 331 | IAELGAF | 0.754 |
| 329 | G | 326 | 332 | AELGAFF | 0.773 |
| 330 | A | 327 | 333 | ELGAFFS | 0.883 |
| 331 | F | 328 | 334 | LGAFFSI | 0.844 |
| 332 | F | 329 | 335 | GAFFSIL | 0.844 |
| 333 | S | 330 | 336 | AFFSILQ | 0.761 |
| 334 | I | 331 | 337 | FFSILQD | 0.876 |
| 335 | L | 332 | 338 | FSILQDM | 0.876 |
| 336 | Q | 333 | 339 | SILQDMR | 0.926 |
| 337 | D | 334 | 340 | ILQDMRN | 0.944 |
| 338 | M | 335 | 341 | LQDMRNT | 1.014 |
| 339 | R | 336 | 342 | QDMRNTI | 0.997 |
| 340 | N | 337 | 343 | DMRNTIM | 0.943 |
| 341 | T | 338 | 344 | MRNTIMA | 0.829 |
| 342 | I | 339 | 345 | RNTIMAS | 0.947 |
| 343 | M | 340 | 346 | NTIMASK | 0.956 |
| 344 | A | 341 | 347 | TIMASKT | 0.87 |
| 345 | S | 342 | 348 | IMASKTV | 0.804 |
| 346 | K | 343 | 349 | MASKTVG | 0.96 |
| 347 | T | 344 | 350 | ASKTVGT | 1.011 |
| 348 | V | 345 | 351 | SKTVGTS | 1.121 |
| 349 | G | 346 | 352 | KTVGTSE | 1.023 |
| 350 | T | 347 | 353 | TVGTSEE | 0.984 |
| 351 | S | 348 | 354 | VGTSEEK | 0.991 |
| 352 | E | 349 | 355 | GTSEEKL | 1.004 |
| 353 | E | 350 | 356 | TSEEKLR | 0.917 |
| 354 | K | 351 | 357 | SEEKLRK | 0.924 |
| 355 | L | 352 | 358 | EEKLRKK | 0.864 |
| 356 | R | 353 | 359 | EKLRKKS | 0.963 |
| 357 | K | 354 | 360 | KLRKKSS | 1.061 |
| 358 | K | 355 | 361 | LRKKSSF | 1.003 |
| 359 | S | 356 | 362 | RKKSSFY | 1.081 |
| 360 | S | 357 | 363 | KKSSFYQ | 1.086 |
| 361 | F | 358 | 364 | KSSFYQS | 1.146 |
| 362 | Y | 359 | 365 | SSFYQSY | 1.164 |
| 363 | Q | 360 | 366 | SFYQSYL | 1.044 |
| 364 | S | 361 | 367 | FYQSYLR | 0.976 |
| 365 | Y | 362 | 368 | YQSYLRR | 1.026 |
| 366 | L | 363 | 369 | QSYLRRT | 1 |
| 367 | R | 364 | 370 | SYLRRTQ | 1 |
| 368 | R | 365 | 371 | YLRRTQS | 1 |
| 369 | T | 366 | 372 | LRRTQSM | 0.923 |
| 370 | Q | 367 | 373 | RRTQSMG | 1.061 |
| 371 | S | 368 | 374 | RTQSMGI | 0.993 |
| 372 | M | 369 | 375 | TQSMGIQ | 0.997 |
| 373 | G | 370 | 376 | QSMGIQL | 0.944 |
| 374 | I | 371 | 377 | SMGIQLG | 1.027 |
| 375 | Q | 372 | 378 | MGIQLGQ | 0.963 |
| 376 | L | 373 | 379 | GIQLGQR | 1.013 |
| 377 | G | 374 | 380 | IQLGQRI | 0.857 |
| 378 | Q | 375 | 381 | QLGQRII | 0.857 |
| 379 | R | 376 | 382 | LGQRIIV | 0.789 |
| 380 | I | 377 | 383 | GQRIIVL | 0.789 |
| 381 | I | 378 | 384 | QRIIVLF | 0.651 |
| 382 | V | 379 | 385 | RIIVLFM | 0.597 |
| 383 | L | 380 | 386 | IIVLFMV | 0.533 |
| 384 | F | 381 | 387 | IVLFMVA | 0.56 |
| 385 | M | 382 | 388 | VLFMVAW | 0.63 |
| 386 | V | 383 | 389 | LFMVAWG | 0.781 |
| 387 | A | 384 | 390 | FMVAWGK | 0.841 |
| 388 | W | 385 | 391 | MVAWGKE | 0.861 |
| 389 | G | 386 | 392 | VAWGKEA | 0.87 |
| 390 | K | 387 | 393 | AWGKEAV | 0.87 |
| 391 | E | 388 | 394 | WGKEAVD | 0.984 |
| 392 | A | 389 | 395 | GKEAVDN | 1.07 |
| 393 | V | 390 | 396 | KEAVDNF | 0.933 |
| 394 | D | 391 | 397 | EAVDNFH | 0.924 |
| 395 | N | 392 | 398 | AVDNFHL | 0.903 |
| 396 | F | 393 | 399 | VDNFHLG | 1.031 |
| 397 | H | 394 | 400 | DNFHLGD | 1.169 |
| 398 | L | 395 | 401 | NFHLGDD | 1.169 |
| 399 | G | 396 | 402 | FHLGDDM | 1.031 |
| 400 | D | 397 | 403 | HLGDDMD | 1.154 |
| 401 | D | 398 | 404 | LGDDMDP | 1.236 |
| 402 | M | 399 | 405 | GDDMDPE | 1.257 |
| 403 | D | 400 | 406 | DDMDPEL | 1.119 |
| 404 | P | 401 | 407 | DMDPELR | 1.046 |
| 405 | E | 402 | 408 | MDPELRT | 0.974 |
| 406 | L | 403 | 409 | DPELRTL | 0.973 |
| 407 | R | 404 | 410 | PELRTLA | 0.859 |
| 408 | T | 405 | 411 | ELRTLAQ | 0.781 |
| 409 | L | 406 | 412 | LRTLAQS | 0.88 |
| 410 | A | 407 | 413 | RTLAQSL | 0.88 |
| 411 | Q | 408 | 414 | TLAQSLI | 0.811 |
| 412 | S | 409 | 415 | LAQSLID | 0.883 |
| 413 | L | 410 | 416 | AQSLIDV | 0.87 |
| 414 | I | 411 | 417 | QSLIDVK | 0.92 |
| 415 | D | 412 | 418 | SLIDVKV | 0.851 |
| 416 | V | 413 | 419 | LIDVKVK | 0.791 |
| 417 | K | 414 | 420 | IDVKVKE | 0.813 |
| 418 | V | 415 | 421 | DVKVKEI | 0.813 |
| 419 | K | 416 | 422 | VKVKEIS | 0.809 |
| 420 | E | 417 | 423 | KVKEISN | 0.96 |
| 421 | I | 418 | 424 | VKEISNQ | 0.956 |
| 422 | S | 419 | 425 | KEISNQE | 0.99 |
| 423 | N | 420 | 426 | EISNQEP | 1.063 |
| 424 | Q | 421 | 427 | ISNQEPL | 1.041 |
| 425 | E | 422 | 428 | SNQEPLK | 1.119 |
| 426 | P | 423 | 429 | NQEPLKL | 0.999 |
